# Routing space exploration for scalable routing in the quantum Internet

**DOI:** 10.1038/s41598-020-68354-y

**Published:** 2020-07-17

**Authors:** Laszlo Gyongyosi, Sandor Imre

**Affiliations:** 1 School of Electronics and Computer Science, University of Southampton, Southampton, SO17 1BJ UK; 2 Department of Networked Systems and Services, Budapest University of Technology and Economics, Budapest, 1117 Hungary; 3 MTA-BME Information Systems Research Group, Hungarian Academy of Sciences, Budapest, 1051 Hungary

**Keywords:** Mathematics and computing, Pure mathematics, Quantum information

## Abstract

The entangled network structure of the quantum Internet formulates a high complexity routing space that is hard to explore. Scalable routing is a routing method that can determine an optimal routing at particular subnetwork conditions in the quantum Internet to perform a high-performance and low-complexity routing in the entangled structure. Here, we define a method for routing space exploration and scalable routing in the quantum Internet. We prove that scalable routing allows a compact and efficient routing in the entangled networks of the quantum Internet.

## Introduction

Quantum information and quantum computations^1–19^ will not only reformulate our view of the nature of computation and communication, but will also open new possibilities for realizing high-performance computer architectures and telecommunication networks^10–17,28–31,33–45,62–77^. Since our traditional data will no longer remain safe in the traditional Internet when quantum computers become available, there will be a need for a fundamentally different network structure: the quantum Internet^20–23,25–27,29,30^.

In a quantum Internet scenario^20–31,43–53,55–61,78–80^, a primary task is to distribute quantum entanglement^54,81–98^ from a source quantum node to a target quantum node through a set of intermediate quantum nodes called quantum repeaters^32,99–112^. The entanglement distribution is realized in a step-by-step manner by the generation of short-distance entangled connections between quantum nodes. Next, the level of entanglement of the entangled connections is increased to generate longer-distance entangled connections. The entanglement level of an entangled connection determines the hop-distance (number of quantum nodes spanned by the particular entangled connection) between a source node and the target node of the given entangled connection. The level increment is realized by the so-called entanglement swapping (entanglement extension) procedure applied in the intermediate quantum repeaters. Specifically, the entanglement distribution is achieved within the framework of the so-called doubling architecture^28,43,44^, where each increment of the level of entanglement doubles the hop-distance. Using the entanglem ent distribution procedure, the distant source node and the target node can share a long-distance entangled connection.

The entangled quantum network structure integrates several entangled paths between a distant source and destination quantum nodes. In a general Internet setting with several legal and transmit users, numerous entangled paths exist in parallel, so the quantum repeaters must process all paths simultaneously. The properties of the entangled paths, along with the internal and external attributes of quantum repeaters (quantum memory usage, auxiliary internal processes and communication between quantum repeaters), formulate an abstracted space, called the routing space, of the quantum Internet. Due to the complex mechanisms of the quantum Internet and to the large number of variables associated with modelling these processes, an efficient method for exploring the routing space of the quantum Internet is essential for high-performance and high-efficiency routing.

A fundamental problem in the quantum Internet is that while routing methods for finding the shortest path in a quantum network are available^28,43,44,46–49,53^, a mathematical model for the working mechanism of the quantum repeaters in the quantum Internet is still missing. While the shortest paths can be determined only with respect to the cost function associated with the quantum links, these models omit the service capabilities and processes of quantum repeaters, which represent a bottleneck in experimental settings. As a corollary, a comprehensive and exhaustive model is required for the description of entanglement distribution and the construction of entangled paths.

Another issue is the lack of scalable routing in the quantum Internet. Scalable routing refers to a routing method that can determine the most appropriate routing mechanism of a particular subnetwork of the quantum Internet. Specifically, scalable routing can decide whether deterministic routing or adaptive routing would be optimal for a given subnetwork. In deterministic routing, the paths between a subset of quantum repeaters are fixed, while in adaptive routing, the paths are selected dynamically in an adaptive manner according to the actual status of the network. The main advantage of deterministic routing is a more compact and faster realization, since it requires no further path selection in a particular subnetwork. However, this is not generally applicable to the whole quantum Internet due to the dynamically changing conditions. However, performance improvement in the quantum Internet is possible if deterministic routing remains are applied in a particular set of subnetworks while quantum network adaptive routing is applied in the remaining parts. Thus, a scaled routing in the quantum Internet would have a more compact structure and be more efficient for path selection in the entangled network.

Here, we define a method for routing space evaluation and for scalable routing in the quantum Internet. The routing space evaluation integrates the derivation of the external and internal characteristics of quantum repeaters and compacts them into a term called the service rate of quantum repeaters. The scalable routing method utilizes the results of routing space exploration to decompose the quantum Internet into subnetworks with deterministic and adaptive routing between the quantum repeaters of the subnetwork. By utilizing the fundaments of queueing theory^113–115^, we define mathematical models for the service rate evaluation of quantum repeaters and entangled paths in the quantum Internet. The routing space exploration method utilizes the developed mathematical models to formulate the routing space that integrates the characteristics of the available paths, the service rates of quantum repeaters and the path service rates between the legal users of the network. Scalable routing results in more efficient routing overall, since it decreases the routing complexity and utilizes the available resources of the quantum Internet more conveniently than does unscaled routing.

The novel contributions of our manuscript are as follows:

1. We define a mathematical model for the service rate evaluation of quantum repeaters and entangled quantum paths in the quantum Internet.
2. We propose a method for routing space exploration of the quantum Internet.
3. We conceal a method for scaled routing in the quantum Internet with deterministic and adaptive routing in the subnetworks.
4. The methods fuse the fundamentals of queueing theory and the theory of quantum networking and entangled networks.

This paper is organized as follows. “System model and problem statement” section presents the system model and the problem statement. “Service rate of a quantum repeater” section defines the service rate evaluation model. “Service rate of a quantum repeater” section proves the evaluation of the service rate of an entangled path. “Routing space exploration and scalable routing” section provides the routing space exploration and scalable routing method. Finally, “Conclusions” section concludes the results. Supplemental information is included in the Appendix.

## System model and problem statement

### System model

The quantum Internet setting is modeled as follows^46^. Let *V* refer to the nodes of an entangled quantum network *N*, with a transmitter quantum node $A\in V$, a receiver quantum node $B\in V$, and quantum repeater nodes $R_i\in V$, $i=1,\ldots ,q$. Let $E=\left\{ E_j\right\}$, $j=1,\ldots ,m$, refer to a set of edges between the nodes of *V*, where each $E_j$ identifies an ${\text {L}}_l$-level entangled connection, $l=1,\ldots ,r$, between quantum nodes $x_j$ and $y_j$ of edge $E_j$, respectively. The entanglement levels of the entangled connections in the entangled quantum network structure are defined as follows.

#### Entanglement levels in the quantum Internet

In a quantum Internet setting, an $N=\left( V,E\right)$ entangled quantum network consists of single-hop and multi-hop entangled connections, such that the single-hop entangled nodes (The *l*-level entangled nodes *x*, *y* refer to quantum nodes *x* and *y* connected by an entangled connection ${{\text {L}}_l}$.) are directly connected through an ${\text {L}}_1$-level entanglement, while the multi-hop entangled nodes communicate through ${\text {L}}_l$-level entanglement. Focusing on the doubling architecture^28–30^ in the entanglement distribution procedure, the number of spanned nodes is doubled in each level of entanglement swapping (entanglement swapping is applied in an intermediate node to create a longer distance entanglement^28^). Therefore, the $d{\left( x,y\right) }_{{\text {L}}_l}$ hop distance in *N* for the ${\text {L}}_l$-level entangled connection between $x,y\in V$ is denoted by^37,46^

1 $$\begin{aligned} d{\left( x,y\right) }_{{\text {L}}_l}=2^{l-1}, \end{aligned}$$

with $d{\left( x,y\right) }_{{\text {L}}_l}-1$ intermediate quantum nodes between *x* and *y*. Therefore, $l=1$ refers to a direct entangled connection between two quantum nodes *x* and *y* without intermediate quantum repeaters, while $l>1$ identifies a multilevel entanglement.

#### Entanglement fidelity

Let

2 $$\begin{aligned} {\left| \beta _{00} \right\rangle } ={\textstyle \frac{1}{\sqrt{2} }} \left( {\left| 00 \right\rangle } +{\left| 11 \right\rangle } \right) \end{aligned}$$

be the target Bell state subject to be created at the end of the entanglement distribution procedure between a particular source node *A* and receiver node *B*. The entanglement fidelity *F* at an actually created noisy quantum system $\sigma$ between *A* and *B* is

3 $$\begin{aligned} F\left( \sigma \right) =\langle {{\beta }_{00}} | \sigma |{{\beta }_{00}} \rangle , \end{aligned}$$

where *F* is a value between 0 and 1, $F=1$ for a perfect Bell state and $F<1$ for an imperfect state^28,30,37^.

#### Routing space

##### **Definition 1**

The ${{\mathrm{S}}}_{\mathfrak {R}}$ routing space of *N* is defined as

4 $$\begin{aligned} {{\mathrm{S}}}_{\mathfrak {R}} \left( N\right) =\left( {{{\mathscr {P}}}}\left( A_{1} \rightarrow B_{1} \right) ,\ldots ,{{\mathscr {P}}}\left( A_{K} \rightarrow B_{K} \right) \right) , \end{aligned}$$

where $${{\mathscr {P}}}\left( A_{i} \rightarrow B_{i} \right) $$ is an entangled path between source user $$A_{i} $$ and destination user $$B_{i} $$, $$i=1,\ldots ,K$$, where *K* is the total number of entangled paths in the quantum network *N*.

An entangled path ${{\mathscr {P}}}\left( A_{i} \rightarrow B_{i} \right)$ is characterized as

5 $$\begin{aligned} {{\mathscr {P}}}\left( A_{i} \rightarrow B_{i} \right) =\left\{ S_{A_{i} \rightarrow B_{i} },\gamma _{i},\Omega _{i},{{\mathscr {S}}}\left( \Omega _{i} \right) \right\} , \end{aligned}$$

where $S_{A_{i} \rightarrow B_{i} }$ is the service rate of ${{\mathscr {P}}}\left( A_{i} \rightarrow B_{i} \right)$ defined as

6 $$\begin{aligned} S_{A_{i} \rightarrow B_{i} } =S\left( A_{i} \right) +\sum _{p=1}^{q}S\left( R_{p} \right) , \end{aligned}$$

where $$S\left( A_{i} \right) $$ is the service rate of source $$A_{i} $$, $$S\left( R_{p} \right) $$ is the service rate of the *p*-th quantum repeater in path $${{\mathscr {P}}}\left( A_{i} \rightarrow B_{i} \right) $$, $$p=1,\ldots ,q$$, *q* is the total number of quantum repeaters in $${{\mathscr {P}}}\left( A_{i} \rightarrow B_{i} \right) $$ [the service rate will be defined in (13)]; $$\gamma _{i} $$, $$\gamma _{i} \le 0$$ is the service rate fluctuation of $${{\mathscr {P}}}\left( A_{i} \rightarrow B_{i} \right) $$ defined as

7 $$\begin{aligned} \gamma _{i} =\gamma \left( A_{i} \right) +\sum _{p=1}^{q}\gamma \left( R_{p} \right) +\gamma \left( B_{i} \right) , \end{aligned}$$

where $$\gamma \left( x\right) $$ of a particular quantum node *x* will be defined in (48), $$\Omega _{i} $$ is the number of available $${{\mathscr {R}}}$$ routes in the quantum Internet for the entanglement distribution from $$A_{i} $$ to $$B_{i} $$, while $${{\mathscr {S}}}\left( \Omega _{i} \right) $$ is a set of the $$\Omega _{i} $$ available routes for $${{\mathscr {P}}}\left( A_{i} \rightarrow B_{i} \right) $$, as

8 $$\begin{aligned} {{\mathscr {S}}}\left( \Omega _{i} \right) =\left( {{\mathscr {R}}}_{1}^{i} ,\ldots ,{{\mathscr {R}}}_{\Omega _{i} }^{i} \right) , \end{aligned}$$

where ${{\mathscr {R}}}_{k}^{i}$ is the *k*-th available route with $S_{A_{i} \rightarrow B_{i} }$ and $\gamma _{i}$, with a shortest route ${{\mathscr {R}}}_{*}^{i}$

9 $$\begin{aligned} {{\mathscr {R}}}_{*}^{i} =\mathop {\max }\limits _{\forall k} {{\mathscr {R}}}_{k}^{i} \left( S_{A_{i} \rightarrow B_{i} } +\gamma _{i} \right) . \end{aligned}$$

Assuming a doubling architecture for the entanglement distribution, an entangled path ${{\mathscr {P}}}\left( A_{i} \rightarrow B_{i} \right)$ consists of $\left| {{\mathscr {P}}}\left( A_{i} \rightarrow B_{i} \right) \right|$ nodes as

10 $$\begin{aligned} \left| {{\mathscr {P}}}\left( A_{i} \rightarrow B_{i} \right) \right| =A_{i} +\sum _{p=1}^{q=d\left( A,B\right) _{{{\mathrm{L}}}_{l} } -1}R_{p} +B_{i}, \end{aligned}$$

where $d\left( A,B\right) _{{{\mathrm{L}}}_{l} } =2^{l-1}$ is the hop-distance between *A* and *B* at an *l*-level entangled connection $L_{l} \left( A,B\right)$ between *A* and *B*.

#### Cycle

##### **Definition 2**

A cycle *C* with cycle-time $${{t}_{C}}={1}/{{{f}_{C}}} \,{\hbox {s}}$$ is set via an oscillator $$O_{C} $$ with frequency $${{f}_{C}}={1}/{{{t}_{C}}}$$ in the quantum nodes used for synchronization of a quantum network.

From Definition 2, *sC* cycles identify $s{{t}_{C}}={s}/{{{f}_{C}}} \,{\hbox {s}}$, where *s* is a nonzero real number.

### Problem statement

The problem statement is given in Problems 1–3.

#### **Problem 1**

*Determine the* $S\left( R\right)$*service rate of all quantum repeaters of the quantum network at a given set of incoming and outcoming entangled connections.*

#### **Problem 2**

*Evaluate the* $S\left( {{\mathscr {P}}}\left( A\rightarrow B\right) \right)$*service rate of an entangled path in the quantum Internet between distant source quantum nodes A and B.*

#### **Problem 3**

*Define the routing space of the quantum Internet (available paths, service rates of quantum repeaters and service rates of the paths). Determine a scaled routing method with deterministic and adaptive routing in particular subnetworks of the quantum Internet.*

The resolutions of Problems 1–3 are given in Theorems 1–3.

## Service rate of a quantum repeater

By utilizing the fundamentals of queueing theory on priority queueing and quantum Shannon theory, we define the service rate of a quantum repeater as follows^113–115^. The system model utilizes a G/G/1 priority queueing model (also referred to as single-server queue with first-in-first-out serving in queueing theory)^113^ for the service rate evaluation of a particular quantum repeater in the quantum Internet. In the proposed G/G/1 setting, the service rates (measured in Bell states per *C*) and the inverse incoming entanglement throughput values (measured in *C* per Bell states) are independent and identically distributed with a general distribution.

Theorem 1 derives the closed-form service rate of a quantum repeater in a G/G/1 setting.

### **Theorem 1**

(Closed-form service rate of a quantum repeater in a G/G/1 setting) *The* $$S\left( R_{i} \left( l_{j},l_{k} \right) \right) $$ *service rate of* *an* *i*-*th quantum repeater* $$R_{i} $$ *can be expressed in a closed-form in a G/G/1 setting, where* $$l_{j} $$ *is an incoming entangled connection of* $$R_{i} $$, *while* $$l_{k} $$ *is the outcoming entangled connection of* $$R_{i} $$.

### Proof

The aim of the proof is to derive the $$S\left( R_{i} \left( l_{j} ,l_{k} \right) \right) $$ service rate of $$R_i$$ in a closed-form. Let $$R_{i} \left( {{\mathscr {S}}}_{l_{in} } \left( R_{i} \right) ,l_{k} \right) $$ refer to an *i*-th quantum repeater node $$R_{i} $$ with a set $${{\mathscr {S}}}_{l_{in} } \left( R_{i} \right) $$ of *p* input entangled connections,

11 $$\begin{aligned} {{\mathscr {S}}}_{l_{in} } \left( R_{i} \right) =\left( l_{in,1},\ldots ,l_{in,p} \right) , \end{aligned}$$

and an output entangled connection $$l_{k} $$.

Then, let $$S\left( R_{i} \left( l_{j},l_{k} \right) \right) $$ be the service rate (measured in Bell states per *C* cycle) of $$R_{i} $$ with incoming entangled connection $$l_{j} $$ and outcoming entangled connection $$l_{k} $$; let $$B_{F} \left( l_{k} \right) $$ be the entanglement throughput (The $$B_{F}$$ entanglement throughput identifies the number of Bell states per cycle of a particular entanglement fidelity *F*.), measured in Bell states per cycle, of the output entangled connection $$l_{k} $$ of $$R_{i} $$.

The optimization problem can be evaluated as a maximization,

12 $$\begin{aligned} c\left( {{\mathscr {P}}}\right) =\max \sum _{R_{i} \in {{\mathscr {P}}}\left( A\rightarrow B\right) }\left( S\left( A\left( l_{k} \right) \right) +S\left( R_{i} \left( l_{j},l_{k} \right) \right) \right) , \end{aligned}$$

where $$S\left( A\left( l_{k} \right) \right) $$ is the service rate of source node *A* with outcoming entangled connection $$l_{k} $$.

Then, by using the G/G/1 priority queueing model, the $S\left( R_{i} \left( l_{j},l_{k} \right) \right)$ service rate for a quantum repeater $R_{i} \left( l_{j},l_{k} \right)$ with a given ${{\mathscr {S}}}_{l_{in} } \left( R_{i} \right) \ne \emptyset$ is defined in a closed-form as

13 $$\begin{aligned} S\left( R_{i} \left( l_{j},l_{k} \right) \right) =\left\{ \begin{array}{ll} \left| B_{F}^{in} \left( l_{j} \right) \right| {\textstyle \frac{2\left( \mu \left( B_{F} \left( l_{k} \right) \right) -\mu \left( B_{F} \left( l_{j} \rightarrow l_{k} \right) \right) \right) }{\alpha _{k} \left( R_{i} \right) \left( \chi _{in}^{2} \left( R_{i} \right) +\chi ^{2} \left( C\left( M\left( R_{i} \left( l_{k} \right) \right) \right) \right) \right) }},&{}\quad {\hbox {if}}\, {{\mathscr {S}}}_{l_{in} } \left( R_{i} \right) \in {{\mathscr {P}}}\left( A\rightarrow B\right) \\ \left| B_{F}^{in} \left( l_{j} \right) \right| {\textstyle \frac{2\left( \mu \left( B_{F} \left( l_{k} \right) \right) -\sum _{i=1}^{g-1}\mu \left( B_{F} \left( l_{i} \rightarrow l_{k} \right) \right) \right) ^{2} }{\omega _{k} \left( R_{i} \right) \left( \chi _{in}^{2} \left( R_{i} \right) +\chi ^{2} \left( C\left( M\left( R_{i} \left( l_{k} \right) \right) \right) \right) \right) }}, &{}\quad {\hbox {if}}\; {{\mathscr {S}}}_{l_{in} } \left( R_{i} \right) \notin {{\mathscr {P}}}\left( A\rightarrow B\right) , \end{array}\right. \end{aligned}$$

where

14 $$\begin{aligned} {\mathrm{2}}\le g\le \left| {{\mathscr {S}}}_{l_{in} } \left( R_{i} \right) \right| , \end{aligned}$$

while $$\left| B_{F}^{in} \left( l_{j} \right) \right| $$ is the number of incoming entangled states (measured in Bell states) in the input entangled connection $$l_{k} $$ of $$R_{i} $$, $${{\mathscr {S}}}_{l_{in} } \left( R_{i} \right) \in {{\mathscr {P}}}\left( A\rightarrow B\right) $$ refers to the situation if the input of $$R_{i} $$ is from a previous node $$R_{h} $$ such that $$R_{h} \in {{\mathscr {P}}}\left( A\rightarrow B\right) $$, and where $${{\mathscr {P}}}\left( A\rightarrow B\right) $$ is a main path between *A* and *B*, and $${{\mathscr {S}}}_{l_{in} } \left( R_{i} \right) \in R_{h} \notin {{\mathscr {P}}}\left( A\rightarrow B\right) $$ refers to the case if the input of $$R_{i} $$ is from a previous node $$R_{h} $$ such that $$R_{h} $$ is not part of the main path, $$R_{h} \notin {{\mathscr {P}}}\left( A\rightarrow B\right) $$.

The terms of $S\left( R_{i} \left( l_{j},l_{k} \right) \right)$ are explained as follows.

The quantity $$\alpha _{k} \left( R_{i} \right) $$ is ratio that models the unavailability of the $$l_{k} $$ of output $$R_{i} $$ as

15 $$\begin{aligned} \alpha _{k} \left( R_{i} \right) ={\textstyle \frac{1}{\mu \left( B_{F} \left( l_{k} \right) \right) }} \sum _{j=1}^{\left| {{\mathscr {S}}}_{l_{in} } \left( R_{i} \right) \right| }\mu \left( B_{F} \left( l_{j} \rightarrow l_{k} \right) \right) , \end{aligned}$$

where $$\mu \left( B_{F} \left( l_{k} \right) \right) $$ is the average entanglement throughput of output entangled connection $$l_{k} $$ of $$R_{i} $$, $$\mu \left( B_{F} \left( l_{j} \rightarrow l_{k} \right) \right) $$ is the average entanglement throughput of the input entangled connection $$l_{j} $$, $$l_{j} \in {{\mathscr {S}}}_{l_{in} } \left( R_{i} \right) $$, $$j=1,\ldots ,\left| {{\mathscr {S}}}_{l_{in} } \left( R_{i} \right) \right| $$, $$\left| {{\mathscr {S}}}_{l_{in} } \left( R_{i} \right) \right| $$ is the cardinality of $${{\mathscr {S}}}_{l_{in} } \left( R_{i} \right) $$, $$\chi _{in}^{2} \left( R_{i} \right) $$ is the coefficient of variation^113–115^ for the *Z* inverse of the $$\omega _{k} \left( R_{i} \right) $$ sum of average entanglement throughput of all incoming entangled connections of connection $$l_{j} $$ in $$R_{i} $$ (measured in *C* per Bell states), as

16 $$\begin{aligned} Z={\textstyle \frac{1}{\omega _{k} \left( R_{i} \right) }}, \end{aligned}$$

where

17 $$\begin{aligned} \omega _{k} \left( R_{i} \right) =\sum _{j=1}^{\left| {{\mathscr {S}}}_{l_{in} } \left( R_{i} \right) \right| }\mu \left( B_{F} \left( l_{j} \rightarrow l_{k} \right) \right) , \end{aligned}$$

thus

18 $$\begin{aligned} \chi _{in}^{2} \left( R_{i} \right) =\left\langle Z^{2} \right\rangle {\textstyle \frac{1}{{\bar{Z}}^{2} }} -1, \end{aligned}$$

where ${\bar{Z}}$ is the average of *Z*,

19 $$\begin{aligned} {\bar{Z}}=\sum _{i=1}^{g}{\textstyle \frac{Z_{i} }{g}}, \end{aligned}$$

where $$g=1$$ if $${{\mathscr {S}}}_{l_{in} } \left( R_{i} \right) \in {{\mathscr {P}}}\left( A\rightarrow B\right) $$, while $$\left\langle Z^{2} \right\rangle $$ is the average of $$Z^{2} $$ as

20 $$\begin{aligned} \left\langle Z^{2} \right\rangle =\sum _{i=1}^{g}{\textstyle \frac{\left( Z_{i} \right) ^{2} }{g}}. \end{aligned}$$

The term $\omega _{k} \left( R_{i} \right)$ in (17) can be rewritten via (15) weighted by the ratio of (15) as

21 $$\begin{aligned} \omega _{k} \left( R_{i} \right) =\alpha _{k} \left( R_{i} \right) \mu \left( B_{F} \left( l_{k} \right) \right) . \end{aligned}$$

Then, $\chi _{in}^{2} \left( R_{i} \right)$ can be set as a constant^113–115^ for all quantum repeaters, $\chi _{in}^{2} \left( R_{i} \right) =\chi _{in}^{2} \left( R\right)$, for $\forall R_{i}$.

The term $$\chi ^{2} \left( C\left( M\left( R_{i} \left( l_{k} \right) \right) \right) \right) $$ is the coefficient of variation^113–115^ of cycles $$C\left( M\left( R_{i} \left( l_{k} \right) \right) \right) $$, where $$C\left( M\left( R_{i} \left( l_{k} \right) \right) \right) $$ characterizes the cycles of the $$M\left( R_{i} \left( l_{k} \right) \right) $$ internal processes of $$R_{i} $$ (quantum memory usage, error correction, purification, etc), will be defined in (37). These cycles reduce the service rate through $$l_{k} $$ of $$R_{i} $$.

The term $\mu \left( B_{F} \left( l_{j} \rightarrow l_{k} \right) \right)$ (average Bell states per *C*) can be rewritten as

22 $$\begin{aligned} \mu \left( B_{F} \left( l_{j} \rightarrow l_{k} \right) \right) =\sum _{A}\sum _{B}\mu \left( B_{F} \left( A\right) \right) \Pr \left( {{\mathscr {P}}}\left( A\rightarrow B\right) \right) {{\mathscr {R}}}\left( AB,R_{i} \left( l_{j},l_{k} \right) \right) , \end{aligned}$$

where $\mu \left( B_{F} \left( A\right) \right)$ is the average output entanglement throughput (Bell states per *C*) of source node *A*, $\Pr \left( {{\mathscr {P}}}\left( A\rightarrow B\right) \right)$ is the probability that a source *A* and a target *B* are connected an entangled path ${{\mathscr {P}}}\left( A\rightarrow B\right)$,

23 $$\begin{aligned} \sum _{A}\sum _{B}\Pr \left( {{\mathscr {P}}}\left( A\rightarrow B\right) \right) =1 , \end{aligned}$$

while ${{\mathscr {R}}}\left( AB,R_{i} \left( l_{j},l_{k} \right) \right)$ is a routing function defined as

24 $$\begin{aligned} {{\mathscr {R}}}\left( AB,R_{i} \left( l_{i},l_{k} \right) \right) =\left\{ \begin{array}{ll} 1, &{}\quad {{\mathrm{if}}}\; R_{i} \in {{\mathscr {P}}}\left( A\rightarrow B\right) \\ 0, &{} {{\mathrm{otherwise}}} \end{array}\right. , \end{aligned}$$

thus the routing function in (24) therefore equals to 1, if quantum repeater $R_{i} \left( l_{i},l_{k} \right)$ is part of the path ${{\mathscr {P}}}\left( A\rightarrow B\right)$, and 0 otherwise.

The $\Phi \left( R_{i} \left( l_{j},l_{k} \right) \right)$ inverse of the service rate $S\left( R_{i} \left( l_{j},l_{k} \right) \right)$ [see (13)] of $R_{i} \left( l_{j},l_{k} \right)$ (measured in *C* per Bell states) is defined as

25 $$\begin{aligned} \Phi \left( R_{i} \left( l_{j},l_{k} \right) \right) =\left\{ \begin{array}{ll} \alpha _{k} \left( R_{i} \right) {\textstyle \frac{\left( \chi _{in}^{2} \left( R_{i} \right) +\chi ^{2} \left( C\left( M\left( R_{i} \left( l_{k} \right) \right) \right) \right) \right) }{\left| B_{F}^{in} \left( l_{j} \right) \right| \left( 2\left( \mu \left( B_{F} \left( l_{k} \right) \right) -\mu \left( B_{F} \left( l_{j} \rightarrow l_{k} \right) \right) \right) \right) }}&{}\quad {\mathrm{if}} \; {{{\mathscr {S}}}}_{l_{in}} \left( R_{i} \right) \in {{\mathscr {P}}}\left( A\rightarrow B\right) \\ \omega _{k} \left( R_{i} \right) {\textstyle \frac{\left( \chi _{in}^{2} \left( R_{i} \right) +\chi ^{2} \left( C\left( M\left( R_{i} \left( l_{k} \right) \right) \right) \right) \right) }{\left| B_{F}^{in} \left( l_{j} \right) \right| 2\left( \mu \left( B_{F} \left( l_{k} \right) \right) -\sum _{i=1}^{g-1}\mu \left( B_{F} \left( l_{i} \rightarrow l_{k} \right) \right) \right) ^{2} }} &{}\quad {\mathrm{if}} \; {{{\mathscr {S}}}}_{l_{in}} \left( R_{i} \right) \notin {{\mathscr {P}}}\left( A\rightarrow B\right) . \end{array}\right. \end{aligned}$$

Using (25), the $f\left( {{\mathscr {P}}}\right)$ objective function subject to a minimization (While the objective function in (12) subject to a maximization utilizes the service rate formula of (13) derived via the G/G/1 priority queueing model, the objective function in (26) utilizes the inverse of (13) and defines a minimization problem.) can be written as

26 $$\begin{aligned} f\left( {{\mathscr {P}}}\right) =\min \sum _{i\in {{\mathscr {P}}}}\left( \Phi \left( A\left( l_{k} \right) \right) +\Phi \left( R_{i} \left( l_{j} ,l_{k} \right) \right) \right) . \end{aligned}$$

The validation of the formula of (13) is as follows.

It can be verified^114,115^, that $\Phi \left( R_{i} \left( l_{j},l_{k} \right) \right)$ [see (25)] can be decomposed as

27 $$\begin{aligned} \Phi \left( R_{i} \left( l_{j},l_{k} \right) \right) =\left\{ \begin{array}{ll} {\textstyle \frac{\psi _{k} \left( R_{i} \right) }{\left| B_{F} \left( l_{j} \right) \right| \left( 1-\varsigma \left( R_{i} \left( l_{j} ,l_{k} \right) \right) \right) }}&{}\quad {\mathrm{if}} \; {{{\mathscr {S}}}}_{l_{in}} \left( R_{i} \right) \in {{\mathscr {P}}}\left( A\rightarrow B\right) \\ {\textstyle \frac{\psi _{k} \left( R_{i} \right) }{\left| B_{F} \left( l_{j} \right) \right| \left( 1-\sum _{z=1}^{g-1}\varsigma \left( R_{i} \left( l_{z},l_{k} \right) \right) \right) }}&{}\quad {\mathrm{if}}\; {{{\mathscr {S}}}}_{l_{in} } \left( R_{i} \right) \notin {{\mathscr {P}}}\left( A\rightarrow B\right) , \end{array}\right. \end{aligned}$$

where $\varsigma \left( R_{i} \left( l_{j},l_{k} \right) \right)$ is defined as a ratio of incoming and outcoming entanglement throughputs

28 $$\begin{aligned} \varsigma \left( R_{i} \left( l_{j},l_{k} \right) \right) ={\textstyle \frac{\mu \left( B_{F} \left( l_{j} \rightarrow l_{k} \right) \right) }{\mu \left( B_{F} \left( l_{k} \right) \right) }}, \end{aligned}$$

while $\psi _{k} \left( R_{i} \right)$ is the number of residual cycles (measured in *C* cycles) defined via (28) as

29 $$\begin{aligned} \psi _{k} \left( R_{i} \right) =\sum _{j=1}^{\left| {{\mathscr {S}}}_{l_{in} } \left( R_{i} \right) \right| }\varsigma \left( R_{i} \left( l_{j} ,l_{k} \right) \right) {\textstyle \frac{\left( \chi _{in}^{2} \left( R_{i} \right) +\chi ^{2} \left( C\left( M\left( R_{i} \left( l_{k} \right) \right) \right) \right) \right) }{2\mu \left( B_{F} \left( l_{k} \right) \right) }}, \end{aligned}$$

where $\chi _{in}^{2} \left( R_{i} \right)$ is given in (18).

The sum of $\sum _{j=1}^{g}\varsigma \left( R_{i} \left( l_{j},l_{k} \right) \right)$ in (29) can be rewritten via (15) as

30 $$\begin{aligned} \begin{aligned} \sum \limits _{j=1}^{\left| {{{\mathscr {S}}}_{{{l}_{in}}}}\left( {{R}_{i}} \right) \right| }{\varsigma \left( {{R}_{i}}\left( {{l}_{j}},{{l}_{k}} \right) \right) }&=\sum \limits _{j=1}^{\left| {{{\mathscr {S}}}_{{{l}_{in}}}}\left( {{R}_{i}} \right) \right| }{\tfrac{\mu \left( {{B}_{F}}\left( {{l}_{j}}\rightarrow {{l}_{k}} \right) \right) }{\mu \left( {{B}_{F}}\left( {{l}_{k}} \right) \right) }} \\&=\tfrac{1}{\mu \left( {{B}_{F}}\left( {{l}_{k}} \right) \right) }\sum \limits _{j=1}^{\left| {{{\mathscr {S}}}_{{{l}_{in}}}}\left( {{R}_{i}} \right) \right| }{\mu \left( \sum \nolimits _{j}{{{B}_{F}}\left( {{l}_{j}}\rightarrow {{l}_{k}} \right) } \right) } \\&=\tfrac{{{\omega }_{k}}\left( {{R}_{i}} \right) }{\mu \left( {{B}_{F}}\left( {{l}_{k}} \right) \right) } \\&=\alpha \left( {{R}_{i}}\left( {{l}_{j}},{{l}_{k}} \right) \right) , \end{aligned} \end{aligned}$$

where $\omega _{k} \left( R_{i} \right)$ is as given in (21).

As follows, (29) can be rewritten as

31 $$\begin{aligned} \psi _{k} \left( R_{i} \right) =\alpha \left( R_{i} \left( l_{j},l_{k} \right) \right) {\textstyle \frac{\left( \chi _{in}^{2} \left( R_{i} \right) +\chi ^{2} \left( C\left( M\left( R_{i} \left( l_{k} \right) \right) \right) \right) \right) }{2\mu \left( B_{F} \left( l_{k} \right) \right) }}. \end{aligned}$$

Thus, using (31), the term in (27) can be rewritten as given in (25), and as a corollary, the formula of (13) is validated.

Next, let study the case if there are multiple possible output paths are available for a given incoming entangled connection. Let $$R_{h} $$ be a source neighbor node of $$R_{i} $$, $$R_{h} \in {{\mathscr {P}}}\left( A,B\right) $$ associated with an incoming entangled connection $$l_{j} $$ of $$R_{i} $$, and let us assume that $$R_{i} $$ has $$\left| {{\mathscr {S}}}_{l_{out} } \left( R_{i} \right) \right| $$ outcoming entangled connections, where $${{\mathscr {S}}}_{l_{out} } \left( R_{i} \right) $$ is the set of *r* output entangled connections of $$R_{i} $$, $${{\mathscr {S}}}_{l_{out} } \left( R_{i} \right) =\left( l_{out,1},\ldots ,l_{out,r} \right) $$.

Using (13), the $$\mu \left( S\left( R_{h} \left( l_{k} \right) \right) \right) $$ average service rate for the output entangled connection $$l_{k} $$ of a particular $$R_{h} $$ from set $${{\mathscr {S}}}_{S} \left( R_{i} \right) $$ can be evaluated as

32 $$\begin{aligned} \mu \left( S\left( R_{h} \left( l_{k} \right) \right) \right) ={\textstyle \frac{1}{\theta \left( R_{h} \left( l_{k} \right) \right) }}, \end{aligned}$$

where $\theta \left( R_{h} \left( l_{k} \right) \right)$ is the first moment of $C\left( M\left( R_{h} \left( l_{k} \right) \right) \right)$ defined as

33 $$\begin{aligned} \theta \left( R_{h} \left( l_{k} \right) \right) =\sum _{k=1}^{\left| {{\mathscr {S}}}_{l_{out} } \left( R_{i} \right) \right| }\Pr \left( R_{i} \left( l_{j} \rightarrow l_{k} \right) \right) \left( \Phi \left( R_{i} \left( l_{j},l_{k} \right) \right) +d\left( R_{i} \right) +\theta \left( R_{i} \left( l_{k} \right) \right) -\zeta \left( R_{i} \right) \right) , \end{aligned}$$

where $$\Phi \left( R_{i} \left( l_{j},l_{k} \right) \right) $$ is evaluated via (27), and $$\Pr \left( R_{i} \left( l_{j} \rightarrow l_{k} \right) \right) $$ is the probability that an incoming entangled state from $$l_{j} $$ of $$R_{i} $$ is distributed through the output $$l_{k} $$ of $$R_{i} $$, evaluated as

34 $$\begin{aligned} \Pr \left( R_{i} \left( l_{j} \rightarrow l_{k} \right) \right) ={\textstyle \frac{\mu \left( B_{F} \left( l_{j} \rightarrow l_{k} \right) \right) }{\omega _{k} \left( R_{i} \right) }}, \end{aligned}$$

where $$\omega _{k} \left( R_{i} \right) $$ is defined in (21); term $$d\left( R_{i} \right) $$ is the sum of additional internal and external *C* cycles related to $$R_{i} $$, as

35 $$\begin{aligned} d\left( R_{i} \right) =C\left( M\left( R_{i} \left( l_{k} \right) \right) \right) +C\left( \Delta \left( R_{h},R_{i} \right) \right) , \end{aligned}$$

where $$C\left( \Delta \left( R_{h},R_{i} \right) \right) $$ is an external term associated to the $$\Delta \left( R_{h},R_{i} \right) $$ transmission process between nodes $$R_{h} $$ and $$R_{i} $$, while $$\zeta \left( R_{i} \right) $$ identifies the cycles of usage of the internal quantum memory of $$R_{i} $$, as

36 $$\begin{aligned} \zeta \left( R_{i} \right) =\left( \left| {{\mathscr {M}}}\left( R_{i} \left( l_{j} \right) \right) \right| +\left| {{\mathscr {M}}}\left( R_{i} \left( l_{k} \right) \right) \right| \right) \times \max \left( C\left( M\left( R_{i} \left( l_{k} \right) \right) ,C\left( \Delta \left( R_{h},R_{i} \right) \right) \right) \right) , \end{aligned}$$

where $$\left| {{\mathscr {M}}}\left( R_{i} \left( l_{j} \right) \right) \right| $$ is the number of entangled states received from $$l_{j} $$ and stored in the quantum memory $${{\mathscr {M}}}$$ of $$R_{i} $$, $$\left| {{\mathscr {M}}}\left( R_{i} \left( l_{k} \right) \right) \right| $$ is the number of entangled states readout from the quantum memory $${{\mathscr {M}}}$$ of $$R_{i} $$ and distributed through connection $$l_{k} $$.

Then, the $\chi ^{2} \left( C\left( M\left( R_{h} \left( l_{k} \right) \right) \right) \right)$ coefficient of variation^113–115^ from $\theta \left( R_{h} \left( l_{k} \right) \right)$ [see (33)] can be evaluated as

37 $$\begin{aligned} \chi ^{2} \left( C\left( M\left( R_{h} \left( l_{k} \right) \right) \right) \right) ={\textstyle \frac{\nu \left( R_{h} \left( l_{k} \right) \right) }{\left( \theta \left( R_{h} \left( l_{k} \right) \right) \right) ^{2} }} -1, \end{aligned}$$

where $\nu \left( R_{h} \left( l_{k} \right) \right)$ is the second moment of $C\left( M\left( R_{h} \left( l_{k} \right) \right) \right)$, as

38 $$\begin{aligned} \nu \left( R_{h} \left( l_{k} \right) \right) =\sum _{k=1}^{\left| {{\mathscr {S}}}_{l_{out} } \left( R_{i} \right) \right| }\Pr \left( R_{i} \left( l_{j} \rightarrow l_{k} \right) \right) \left( \Phi \left( R_{i} \left( l_{j},l_{k} \right) \right) +d\left( R_{i} \right) +\theta \left( R_{i} \left( l_{k} \right) \right) -\zeta \left( R_{i} \right) \right) ^{2}. \end{aligned}$$

Then, using (37), the term $\Phi \left( R_{h} \left( l_{k} \right) \right)$ can be evaluated via (25).

The proof is concluded here. □

**Figure 1:**
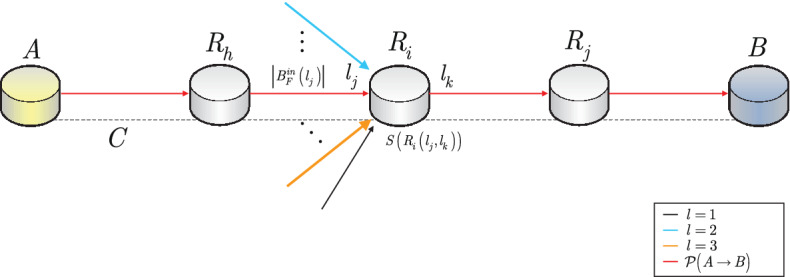
A network situation in a quantum Internet setting with an *i*-th quantum repeater $R_i$ with service rate $S\left( R_i\left( l_j,l_k\right) \right)$ in a main path ${\mathscr {P}}\left( A\rightarrow B\right)$ between a source quantum node *A* and receiver quantum node *B*. The previous neighbour of $R_i$ is $R_h$ and the next neighbour of $R_i$ is $R_j$. The $R_i$ node has an incoming entangled connection $l_j$ from the main path with $\left| B^{in}_F\left( l_j\right) \right|$ incoming Bell states, and with $\left| {{\mathscr {S}}}_{l_{in}}\left( R_i\right) \right| -1$ other incoming entangled connections from other nodes, where $\left| {{\mathscr {S}}}_{l_{in}}\left( R_i\right) \right|$ is the number of incoming entangled connections and an outcoming entangled connection $l_k$. The entangled connections have different *l* levels of entanglement (depicted by different colours: the entangled connections of the main path are denoted by red arrows). A *C* cycle in the quantum network is set via an $O_C$ oscillator in the quantum nodes (depicted by a dashed grey line), $t_C={1}/{f_C}$, at a particular oscillator frequency $f_C$.

Figure 1 depicts the proposed system model for the service rate evaluation of a quantum repeater.

## Service rate of entangled paths

Theorem 2 derives the closed-form service rate of an entangled path in a G/G/1 setting, at a doubling architecture.

### **Theorem 2**

(Closed-form service rate of an entangled path) *The* $S\left( {{\mathscr {P}}}\left( A\rightarrow B\right) \right)$ *service rate of an entangled path* ${{\mathscr {P}}}\left( A\rightarrow B\right)$ *between distant quantum nodes* *A* *and* *B* *at a doubling architecture is* $S\left( {\mathscr {P}}\left( A\rightarrow B \right) \right) ={1}/{\left( {{\Phi }_{l=1}}\left( A,{{R}_{1}} \right) +\sum \nolimits _{i=2}^{q-2}{{{\Phi }_{l=1}}\left( {{R}_{i}},{{R}_{i+1}} \right) }+{{\Phi }_{l=1}}\left( {{R}_{q}},B \right) +\Upsilon \left( {{U}_{swap}} \right) \right) }$, *where* *q* *is the total number of quantum repeaters in* ${{\mathscr {P}}}\left( A\rightarrow B\right)$, $\Phi _{l=1} \left( x,y\right) =\Phi \left( x\right) +\Phi \left( y\right) +C\left( M\left( x\right) \right) +C\left( M\left( y\right) \right) +C\left( \Delta \left( x,y\right) \right)$, *x* *and* *y* *are* $l=1$ *level entangled source and target quantum nodes connected by* ${{\mathrm{L}}}_{1} \left( x,y\right)$, *while* $\Upsilon \left( U_{swap}\right)$ *is the service rate decrement in the entanglement distribution caused by the* $U_{swap}$ *entanglement swapping operation*.

### Proof

Let $$R_{S} $$ and $$R_{D} $$ be two quantum repeaters connected by an $$l=1$$ level entangled connection $${{\mathrm{L}}}_{1} \left( x,y\right) $$, with service rates $$S\left( {{R}_{S}} \right) $$ and $$S\left( {{R}_{D}} \right) $$ determined via Theorem 1.

Then, the $$S_{l=1} \left( R_{S},R_{D} \right) $$ service rate between $$R_{S} $$ and $$R_{D} $$ is as

39 $$\begin{aligned} \begin{aligned} {{S}_{l=1}}\left( {{R}_{S}},{{R}_{D}} \right)&=\tfrac{1}{{{\Phi }_{l=1}}\left( {{R}_{S}},{{R}_{D}} \right) } \\&=\tfrac{1}{\Phi \left( {{R}_{S}} \right) +\Phi \left( {{R}_{D}} \right) +C\left( M\left( {{R}_{S}} \right) \right) +C\left( M\left( {{R}_{D}} \right) \right) +C\left( \Delta \left( {{R}_{S}},{{R}_{D}} \right) \right) } \\&=S\left( {{R}_{S}} \right) +S\left( {{R}_{D}} \right) +\xi \left( {{R}_{S}},{{R}_{D}} \right) , \end{aligned} \end{aligned}$$

where term $\xi \left( R_{S},R_{D} \right) \le 0$ refers to service rate degradation, defined as

40 $$\begin{aligned} \xi \left( R_{S},R_{D} \right) ={\textstyle \frac{\Phi \left( R_{S} \right) \Phi \left( R_{D} \right) -\left( \left( \Phi \left( R_{D} \right) +\Phi \left( R_{S} \right) \right) \left( \Phi \left( R_{S} \right) +\Phi \left( R_{D} \right) +C\left( M\left( R_{S} \right) \right) +C\left( M\left( R_{D} \right) \right) +C\left( \Delta \left( R_{S},R_{D} \right) \right) \right) \right) }{\left( \Phi \left( R_{S} \right) +\Phi \left( R_{D} \right) +C\left( M\left( R_{S} \right) \right) +C\left( M\left( R_{D} \right) \right) +C\left( \Delta \left( R_{S},R_{D} \right) \right) \right) \Phi \left( R_{S} \right) \Phi \left( R_{D} \right) }}, \end{aligned}$$

where the simplified notations of $\Phi \left( R_{i} \right)$ and $C\left( M\left( R_{i} \right) \right)$ are used for $\Phi \left( R_{i} \left( l_{j},l_{k} \right) \right)$ and $C\left( M\left( R\left( l_{k} \right) \right) \right)$, respectively.

Let assume that *N* is utilized via the doubling architecture, with hop-distance $d\left( x,y\right) _{{{\mathrm{L}}}_{l} } =2^{l-1}$ for an ${{\mathrm{L}}}_{l}$-level entangled connection between quantum nodes *x* and *y*. Then, for a source *A* and destination *B*, the entanglement distribution process and the generation of the ${{\mathscr {P}}}\left( A\rightarrow B\right)$ entangled path is characterized via the $S\left( {{\mathscr {P}}}\left( A\rightarrow B\right) \right)$ service rate (The formula of (41) assumes a path in a doubling architecture with a source node *A*, destination node *B*, and with *q* intermediate quantum repeaters. The formula defines the inverse of the sum of service rate inverses—service rate inverse is given in (25)—taken for the total $q+2$ nodes of the path, amended by a $\Upsilon \left( U_{swap}\right)$ residual quantity (42) that identifies a service rate decrement caused by entanglement swapping in the nodes.), as

41 $$\begin{aligned} S\left( {{\mathscr {P}}}\left( A\rightarrow B\right) \right) ={\textstyle \frac{1}{\Phi _{l=1} \left( A,R_{1} \right) +\sum _{i=2}^{q-2}\Phi _{l=1} \left( R_{i},R_{i+1} \right) +\Phi _{l=1} \left( R_{q},B\right) +\Upsilon \left( U_{swap}\right) }}, \end{aligned}$$

where *q* is the total number of quantum repeaters of ${{\mathscr {P}}}\left( A\rightarrow B\right)$, $q-2$ is the total number of intermediate quantum repeater pairs on the path excluding the boundary nodes, $\Upsilon \left( U_{swap}\right)$ is the service rate decrement (measured in *C* cycles) in the entanglement distribution caused by the $U_{swap}$ entanglement swapping operations

42 $$\begin{aligned} \Upsilon \left( U_{swap}\right) =\sum _{i=1}^{n_{swap} =d\left( A,B\right) _{{{\mathrm{L}}}_{l} } -1}C\left( R_{i}^{swap} \right) , \end{aligned}$$

where $$n_{swap} $$ is the number of entanglement swapping operations required for an $${{\mathrm{L}}}_{l} $$-level entangled connection between distant *A* and *B*,

43 $$\begin{aligned} n_{swap} =d\left( A,B\right) _{{{\mathrm{L}}}_{l} } -1, \end{aligned}$$

while $$C\left( R_{i}^{swap} \right) $$ is the cycles required for the entanglement swapping an *i*-th swapping quantum repeater $$R_{i}^{swap} $$. □

### Path service rate algorithm

Using (41), a cumulative service rate $S_{\Sigma } \left( {{\mathscr {P}}}\left( A\rightarrow B\right) \right)$ can be evaluated for all source *A* and destination *B* in the network *N* as

44 $$\begin{aligned} S_{\Sigma } \left( {{\mathscr {P}}}\left( A\rightarrow B\right) \right) =\sum _{A}\sum _{B}\Pr \left( {{\mathscr {P}}}\left( A\rightarrow B\right) \right) S\left( {{\mathscr {P}}}\left( A\rightarrow B\right) \right) , \end{aligned}$$

where $\Pr \left( {{\mathscr {P}}}\left( A\rightarrow B\right) \right)$ is the probability of an entangled path ${{\mathscr {P}}}\left( A\rightarrow B\right)$ between *A* and *B*, and $\sum _{A}\sum _{B}\Pr \left( {{\mathscr {P}}}\left( A\rightarrow B\right) \right) =1$.

The steps are detailed in Algorithm 1. The algorithm utilizes the proposed system parameterization for a given path $${{\mathscr {P}}}\left( A\rightarrow B\right) $$ between source node *A* and target node *B*, with *q* intermediate quantum repeaters. The algorithm evaluates $$\mu \left( B_{F} \left( l_{i} \rightarrow l_{k} \right) \right) $$ via (22), the coefficient $$\omega _{k} \left( R_{j} \right) $$ via (21), determines $$\Pr \left( l\left( R_{i} \left( l_{j} \right) , R_{i} \left( l_{k} \right) \right) \right) $$ via (34), evaluates $$\theta \left( R_{j} \left( l_{k} \right) \right) $$ via (33), $$\nu \left( R_{j} \left( l_{k} \right) \right) $$ via (38), and $$\chi ^{2} \left( M\left( R_{i} \left( l_{j},l_{k} \right) \right) \right) $$ via (37), along with the determination of the cycle reduction via the usage of the internal quantum memory $$\varsigma \left( R_{i} \left( l_{j} ,l_{k} \right) \right) $$ by (28), and $$\psi _{k} \left( R_{i} \right) $$ via (29). Finally, the $$S\left( R_{i} \left( l_{j},l_{k} \right) \right) $$ service rates of node $$R_{i} $$ with incoming entangled connection $$l_{j} $$ and outcoming entangled connection $$l_{k} $$ are determined using (13) for all nodes of the path, and outputs $$S\left( {{\mathscr {P}}}\left( A\rightarrow B\right) \right) $$ of the entangled path $${{\mathscr {P}}}\left( A\rightarrow B\right) $$ via (41).

**Table:** 

**Algorithm 1** Service rate of a path	
**Input**: Parameterization of path $${{\mathscr {P}}}\left( A\rightarrow B\right) $$ between source node *A* and target node *B*, with *q* intermediate quantum repeaters. **Output**: Service rate $$S\left( {{\mathscr {P}}}\left( A\rightarrow B\right) \right) $$ of $${{\mathscr {P}}}\left( A\rightarrow B\right) $$. **Step 1**. Set the *C* cycle via oscillator $$O_{C} $$ used in the quantum nodes of the quantum network *N*. **Step 2**. Parameterize the quantum network via $$\mu \left( B_{F} \left( A\right) \right) $$, $$\chi _{in}^{2} \left( A\right) $$, $$\Pr \left( l\left( A,B\right) \right) $$ for a source node *A* and target node *B*, and via $$\mu \left( B_{F} \left( l_{k} \right) \right) $$, and $$C\left( M\left( R_{i} \left( l_{k} \right) \right) \right) $$ for an $$R_{i} $$ *i*-th quantum repeater, $$i=1,\ldots ,q$$. **Step 3**. Determine $$\mu \left( B_{F} \left( l_{i} \rightarrow l_{k} \right) \right) $$ via (22), and $$\omega _{k} \left( R_{j} \right) $$ via (21). **Step 4**. Evaluate $$\Pr \left( l\left( R_{i} \left( l_{j} \right) ,R_{i} \left( l_{k} \right) \right) \right) $$ via (34). **Step 5**. Determine $$\theta \left( R_{j} \left( l_{k} \right) \right) $$ via (33), $$\nu \left( R_{j} \left( l_{k} \right) \right) $$ via (38), and $$\chi ^{2} \left( M\left( R_{i} \left( l_{j},l_{k} \right) \right) \right) $$ via (37). **Step 6**. Evaluate the cycle reduction via the usage of the internal quantum memory $$\varsigma \left( R_{i} \left( l_{j},l_{k} \right) \right) $$ by (28), and $$\psi _{k} \left( R_{i} \right) $$ via (29). **Step 7**. Compute the $$S\left( R_{i} \left( l_{j},l_{k} \right) \right) $$ service rate of node $$R_{i} $$ with incoming entangled connection $$l_{j} $$ and outcoming entangled connection $$l_{k} $$ via (13). **Step 8**. Output $$S\left( {{\mathscr {P}}}\left( A\rightarrow B\right) \right) $$ of the entangled path $${{\mathscr {P}}}\left( A\rightarrow B\right) $$ via (41).	

## Routing space exploration and scalable routing

Theorem 3 derives the optimal routing for the subnetworks, using the closed-form service rate formulas of Theorems 1–2. The derivations utilize a G/G/1 setting and a doubling architecture.

### **Theorem 3**

(Scalable routing in the quantum Internet) *An* ${{\mathscr {R}}}_{S} \left( N\right)$ *scaled routing function for the quantum Internet can be determined as* ${{\mathscr {R}}}_{S} \left( N\right) =p_{d} {{\mathscr {R}}}_{d} +p_{a} {{\mathscr {R}}}_{a}$, *where* ${{p}_{d}}={\left| {{{\mathscr {S}}}_{{{{\mathscr {R}}}_{d}}}} \right| }/{\left| {{V}_{R}} \right| }$ *and* ${{p}_{a}}={\left| {{{\mathscr {S}}}_{{{{\mathscr {R}}}_{a}}}} \right| }/{\left| {{V}_{R}} \right| }$ *are the probabilities of* ${{\mathscr {R}}}_{d}$ *deterministic routing and* ${{\mathscr {R}}}_{a}$ *adaptive routing in the network* *N*, $p_{a} +p_{d} =1$, *while* $\left| V_{R} \right| =\left| {{\mathscr {S}}}_{{{\mathscr {R}}}_{a} } \right| +\left| {{\mathscr {S}}}_{{{\mathscr {R}}}_{d} } \right|$ *and* $\left| \cdot \right|$ *is the cardinality*, ${{\mathscr {S}}}_{{{\mathscr {R}}}_{d} }$ *is a set of subnetworks in which* ${{\mathscr {R}}}_{d}$ *deterministic routing can be applied*, ${{\mathscr {S}}}_{{{\mathscr {R}}}_{d} } ={{\mathscr {S}}}_{{{\mathscr {R}}}_{d,1} } \bigcup \cdots \bigcup {{\mathscr {S}}}_{{{\mathscr {R}}}_{d,{{\mathscr {D}}}} }$, *where* ${{\mathscr {D}}}$ *is the number of subsets*, *and* ${{\mathscr {S}}}_{{{\mathscr {R}}}_{a} }$ *is a set of subnetworks in which* ${{\mathscr {R}}}_{a}$ *adaptive routing can be applied*, ${{\mathscr {S}}}_{{{\mathscr {R}}}_{a} } ={{\mathscr {S}}}_{{{\mathscr {R}}}_{a,1} } \bigcup \cdots \bigcup {{\mathscr {S}}}_{{{\mathscr {R}}}_{a,{{\mathscr {A}}}} }$, *where* ${{\mathscr {A}}}$ *is the number of subsets*.

### Proof

The proof includes a predictive method for routing space exploration in the quantum Internet. The method utilizes the properties of the quantum nodes, such as transmission between the nodes, and also integrates an updating mechanism motivated by machine learning approaches^116–118^ to find the highest service rate path in the quantum network. The scalable routing function is derived via Algorithm 2.

Let *A* and *B* be the source and target quantum nodes. Then, let $$S\left( R_{i} \right) $$ refer to the service rate of $$R_{i} $$ as defined in (13). Let $$R_{j} $$ be a neighbor node connected by an entangled connection with $$R_{i} $$, and let $$R_{k} $$ be a next neighbor of $$R_{j} $$, with service rates $$S\left( R_{j} \right) $$ and $$S\left( R_{k} \right) $$, respectively.

Let $$R_{j} \left( W_{R_{k} \rightarrow B} \right) $$ be the maximal weighted service rate $$S_{R_{k} \rightarrow B} $$ from the *k*-th quantum repeater to the destination *B* evaluated in the *j*-th node $$R_{j} $$, as

45 $$\begin{aligned} R_{j} \left( W_{R_{k} \rightarrow B} \right) =\mathop {\max }\limits _{R_{n} \in {{\mathscr {S}}}_{N} \left( R_{j} \right) } \left( W_{R_{n} \rightarrow B} \right) , \end{aligned}$$

where $${{\mathscr {S}}}_{N} \left( R_{j} \right) $$ is the set of next (i.e., toward destination) quantum nodes that share entangled connection with $$R_{j} $$, $$R_{k} \in {{\mathscr {S}}}_{N} \left( R_{j} \right) $$, while $$W_{R_{n} \rightarrow B} $$ is the weighted service rate from $$R_{n} $$ to *B*, defined as

46 $$\begin{aligned} W_{R_{n} \rightarrow B} =\sum _{p=1}^{V_{R_{n} \rightarrow B} }S\left( R_{p} \right) +\gamma \left( R_{p} \right) , \end{aligned}$$

where $$V_{R_{n} \rightarrow B} $$ is the number of quantum repeaters of path $${{\mathscr {P}}}\left( R_{n} \rightarrow B\right) $$ from $$R_{n} $$ to *B*, defined as

47 $$\begin{aligned} V_{R_{n} \rightarrow B} =\left| {{\mathscr {P}}}\left( R_{n} \rightarrow B\right) \right| -1, \end{aligned}$$

where $$\left| {{\mathscr {P}}}\left( R_{n} \rightarrow B\right) \right| $$ is refers to the total number of nodes of path $${{\mathscr {P}}}\left( R_{n} \rightarrow B\right) $$, while $$\gamma \left( R_{p} \right) \le 0$$ is the entanglement throughput reduction associated with $$R_{p} $$, defined as

48 $$\begin{aligned} \gamma \left( R_{p} \right) ={\textstyle \frac{\Phi \left( R_{p} \right) -\left( \Phi \left( R_{p} \right) +C\left( M\left( R_{p} \right) \right) +C\left( \Delta \left( R_{p},R_{p+1} \right) \right) \right) }{\left( \Phi \left( R_{p} \right) +C\left( M\left( R_{p} \right) \right) +C\left( \Delta \left( R_{p} ,R_{p+1} \right) \right) \right) \Phi \left( R_{p} \right) }}, \end{aligned}$$

where $$C\left( \Delta \left( R_{p},R_{p+1} \right) \right) $$ is a delay between $$R_{p} $$ and $$R_{p+1} $$, ($$R_{V_{R_{n} \rightarrow B} +1} =B$$); in (48) the simplified notations of $$\Phi \left( R_{i} \right) $$ and $$C\left( M\left( R_{i} \right) \right) $$ are used for $$\Phi \left( R_{i} \left( l_{j},l_{k} \right) \right) $$ and $$C\left( M\left( R\left( l_{k} \right) \right) \right) $$, respectively.

**Figure 2:**
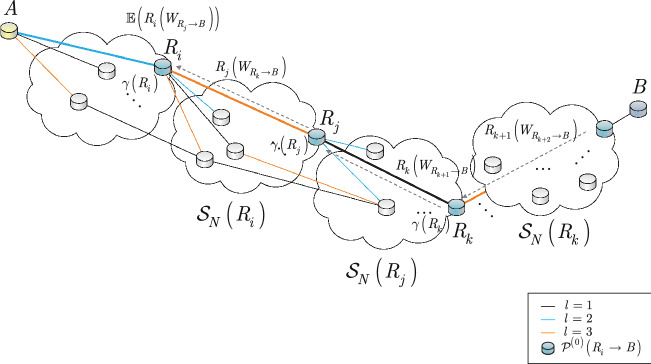
Routing space exploration in the quantum Internet. This method evaluates the service rates of paths between a source quantum repeater $$R_{i} $$ and target node *B*; $$\gamma $$ is the entanglement throughput reduction associated with the nodes and coefficients $$\Omega $$, $${{\mathscr {S}}}\left( \Omega \right) $$ and $${{\mathscr {R}}}_{*} $$ for a given $${{\mathscr {P}}}\left( A\rightarrow B\right) $$. An *i*-th quantum repeater $$R_{i} $$ has a next neighbour set $${{\mathscr {S}}}_{N} \left( R_{i} \right) $$ (depicted by a cloud) with a set of neighbouring quantum repeaters. From each $${{\mathscr {S}}}_{N} \left( R_{i} \right) $$, a particular quantum repeater $$R_{j} $$ is selected such that the $$W_{R_{j} \rightarrow B} $$ weighted service rate from $$R_{j} $$ to *B* is maximized (the quantum repeaters of the initial path are depicted by green nodes). The $$R_{k} \left( W_{R_{k+1} \rightarrow B} \right) $$ service rate information is backpropagated from $$R_{k} $$ to $$R_{j} $$ as side information, and $$R_{j} \left( W_{R_{k} \rightarrow B} \right) $$ is backpropagated from $$R_{j} $$ to $$R_{i} $$ to update estimation $${\mathbb {E}}\left( R_{i} \left( W_{R_{j} \rightarrow B} \right) \right) $$ (classical links are depicted by grey dashed lines).

By propagating backward (see Fig. 2) the value of $$R_{j} \left( W_{R_{k} \rightarrow B} \right) $$ [see (45)] to node $$R_{i} $$, node $$R_{i} $$ can determine the estimation $${\mathbb {E}}\left( R_{i} \left( W_{R_{j} \rightarrow B} \right) \right) $$ as (A backpropagation method is also used in Q-learning based routing methods^117, 118^.)

49 $$\begin{aligned} {\mathbb {E}}\left( R_{i} \left( W_{R_{j} \rightarrow B} \right) \right) =R_{j} \left( W_{R_{k} \rightarrow B} \right) +\left( S\left( R_{j} \right) +\gamma \left( R_{j} \right) \right) , \end{aligned}$$

Using the side information available, an algorithm can be defined to determine the routing space of the quantum Internet. The details are given in Algorithm 2.

The proof concludes here. □

Figure 2 shows the procedure for determining the service rates of the paths. The initial path between an *i*-th quantum repeater $$R_{i} $$ and the target node *B* is $${{\mathscr {P}}}^{\left( 0\right) } \left( R_{i} \rightarrow B\right) $$.

### Routing space exploration algorithm

Algorithm 2 utilizes local and nonlocal information for the determination of the service rate of a particular path. The algorithm uses the proposed service rates formulas of quantum nodes of *N*, and evaluates the related coefficients. The algorithm determines the $$W_{R_{i} \rightarrow B} $$ maximized weighted service rate between $$R_{i} $$ and *B* in an iterative manner. The algorithm also utilizes an $$\ell $$ learning rate coefficient in the parameter updating mechanism. The algorithm evaluates $$S_{A\rightarrow B} $$, $$\Omega $$, $${{\mathscr {S}}}\left( \Omega \right) $$ [see (8)] and $${{\mathscr {R}}}_{*} $$ [see (9)] of path $${{\mathscr {P}}}\left( A\rightarrow B\right) $$, for all paths $${{\mathscr {P}}}\left( A_{i} \rightarrow B_{i} \right) $$, $$i=1,\ldots ,K$$. In the internal steps, it evaluates $$\gamma _{i} $$ via (7), along with the number $$\Omega _{i} $$ of available routes for $${{\mathscr {P}}}\left( A_{i} \rightarrow B_{i} \right) $$, determines $${{\mathscr {S}}}\left( \Omega _{i} \right) $$ via (8), and $${{\mathscr {R}}}_{*}^{i} $$ via in (9). Finally, the algorithm outputs the routing space $${{\mathrm{S}}}_{\mathfrak {R}} \left( N\right) $$ via (4).

**Table:** 

**Algorithm 2** Routing space exploration	
**Input**: Service rates of quantum nodes of *N*. **Output**: Routing space $${{\mathrm{S}}}_{\mathfrak {R}} \left( N\right) $$ of *N*. **Step 1**. Let $$R_{i} $$ and $$R_{j} $$ be quantum repeaters with service rates $$S\left( R_{j} \right) $$ and $$S\left( R_{k} \right) $$ evaluated via (13) and let $${{\mathscr {P}}}^{\left( 0\right) } \left( R_{i} \rightarrow B\right) $$ be an initial path from $$R_{i} $$ to *B* with $$V_{R_{i} \rightarrow B} =\left| {{\mathscr {P}}}^{\left( 0\right) } \left( R_{i} \rightarrow B\right) \right| -1$$ quantum repeaters. Set the $$R_{i}^{\left( 0\right) } \left( W_{R_{j} \rightarrow B} \right) $$ initial value in $$R_{i} $$ for the $$W_{R_{j} \rightarrow B} $$ weighted service rate (46) from to $$R_{i} $$ to *B*, as $$R_{i}^{\left( 0\right) } \left( W_{R_{j} \rightarrow B} \right) =W_{R_{j} \rightarrow B}^{\left( 0\right) }$$, where $$W_{R_{j} \rightarrow B}^{\left( 0\right) } $$ is the initial value of the weighted service rate in $$R_{i} $$, as $$W_{R_{j} \rightarrow B}^{\left( 0\right) } =\sum _{p=1}^{V_{R_{j} \rightarrow B} }S^{\left( 0\right) } \left( R_{p} \right) +\gamma ^{\left( 0\right) } \left( R_{p} \right) $$, and $$S^{\left( 0\right) } \left( R_{p} \right) $$ is determined via (13), while $$\gamma ^{\left( 0\right) } \left( R_{p} \right) $$ is via (48) for all quantum repeaters. **Step 2**. In $$R_{j} $$, evaluate $$R_{j} \left( W_{R_{k} \rightarrow B} \right) $$ via the maximization of (45) for all neighbors of $$R_{j} $$,$$\forall R_{n} \in {{\mathscr {S}}}_{N} \left( R_{j} \right) $$. **Step 3**. Propagate back $$R_{j} \left( W_{R_{k} \rightarrow B} \right) $$ to $$R_{i} $$, and update $$R_{i}^{\left( 0\right) } \left( W_{R_{j} \rightarrow B} \right) $$ (50) via estimation $${\mathbb {E}}\left( R_{i} \left( W_{R_{j} \rightarrow B} \right) \right) $$ (49) to $$R_{i} \left( W_{R_{j} \rightarrow B} \right) $$ as $$R_{i} \left( W_{R_{j} \rightarrow B} \right) =R_{i}^{\left( 0\right) } \left( W_{R_{j} \rightarrow B} \right) +\ell \left( {\mathbb {E}}\left( R_{i} \left( W_{R_{j} \rightarrow B} \right) \right) -R_{i}^{\left( 0\right) } \left( W_{R_{j} \rightarrow B} \right) \right) $$, where $${\mathbb {E}}\left( R_{i} \left( W_{R_{j} \rightarrow B} \right) \right) =R_{j} \left( W_{R_{k} \rightarrow B} \right) +\left( S\left( R_{j} \right) +\gamma \left( R_{j} \right) \right) ,$$ and $$\ell $$ is the learning rate, $$\ell \in \left[ 0,1\right] $$. **Step 4**. Repeat step 3 to for all neighbors of $$R_{i} $$, $$\forall R_{n} \in {{\mathscr {S}}}_{N} \left( R_{i} \right) $$. **Step 5**. Output the $$W_{R_{i} \rightarrow B} $$ maximized weighted service rate between $$R_{i} $$ and *B* as $$W_{R_{i} \rightarrow B} =\mathop {\max }\limits _{R_{n} \in {{\mathscr {S}}}_{N} \left( R_{i} \right) } \left( R_{n} \left( W_{R_{j} \rightarrow B} \right) \right) $$, where $$R_{n} \left( W_{R_{j} \rightarrow B} \right) $$ is determined via (52). **Step 6**. Repeat the procedure until source node *A* to output $$S_{A\rightarrow B} $$, $$\Omega $$, $${{\mathscr {S}}}\left( \Omega \right) $$ [see (8)] and $${{\mathscr {R}}}_{*} $$ [see (9)] of path $${{\mathscr {P}}}\left( A\rightarrow B\right) $$. **Step 7**. Repeat the steps for all paths $${{\mathscr {P}}}\left( A_{i} \rightarrow B_{i} \right) $$, $$i=1,\ldots ,K$$. Determine $$\gamma _{i} $$ via (7), the number $$\Omega _{i} $$ of available routes for $${{\mathscr {P}}}\left( A_{i} \rightarrow B_{i} \right) $$, $${{\mathscr {S}}}\left( \Omega _{i} \right) $$ via (8), and $${{\mathscr {R}}}_{*}^{i} $$ via in (9). **Step 8**. Output routing space $${{\mathrm{S}}}_{\mathfrak {R}} \left( N\right) $$ via (4).	(50) (51) (52) (53)

### Routing scaling algorithm

Using the results of Algorithm 2 for determining the service rates of paths, the neighbouring quantum repeaters can be selected from each quantum repeater to establish scalable routing. The routing scaling algorithm uses the path service rate information to find the entangled path ${{\mathscr {P}}}^{*}$ with the highest weighted service rate $W_{A\rightarrow B}$, and uses ${{\mathscr {R}}}_{d}$ deterministic routing if the service rate degradation coefficient $\xi \left( R_{i},R_{j} \right)$ [see (40)] between a particular source and destination quantum repeater in ${{\mathscr {P}}}^{*}$ is below a critical threshold value $\partial S^{*}$; otherwise, it uses ${{\mathscr {R}}}_{a}$ adaptive routing between the quantum nodes.

Algorithm 3 provides the steps of the routing scaling. The algorithm outputs a highest service path $${{\mathscr {P}}}^{*} $$ with a scaled routing function $${{\mathscr {R}}}_{S} \left( N\right) $$ for quantum network *N*. The algorithm utilizes the parameterized routing space $${{\mathrm{S}}}_{\mathfrak {R}} \left( N\right) $$ of *N* outputted via Algorithm 2. As a main contribution of the algorithm, it evaluates $$S_{l=1} \left( R_{i},R_{j} \right) $$ between $$R_{i} $$ and $$R_{j} $$ via (39) as $$S_{l=1} \left( R_{i},R_{j} \right) =S\left( R_{i} \right) +S\left( R_{j} \right) +\xi \left( R_{i} ,R_{j} \right) $$, where $$S\left( R_{i} \right) $$ and $$S\left( R_{j} \right) $$ are determined via (13), while $$\xi \left( R_{i},R_{j} \right) $$ is as in (40). Then, it makes a decision, using the relation of $$\left| \xi \left( R_{i} ,R_{j} \right) \right| <\partial S^{*} $$. If the relation is true, then it sets a $${{\mathscr {R}}}_{d} $$ deterministic routing between $$R_{i} $$ and $$R_{j} $$. Otherwise, the algorithm sets a $${{\mathscr {R}}}_{a} $$ adaptive routing between $$R_{i} $$ and $$R_{j} $$. After some internal steps and calculations, the algorithm determines $${{\mathscr {P}}}^{*} \left( R_{S} \rightarrow R_{D} \right) $$ for all $$R_{S} $$ source and $$R_{D} $$ destinations, and it establishes the selection of the appropriate routing method for all $$\left\{ R_{i},R_{j} \right\} $$ quantum repeater pairs of path $${{\mathscr {P}}}^{*} \left( R_{S} \rightarrow R_{D} \right) $$. Finally, the algorithm determines the appropriate routing mechanism for all sub-networks of the particular quantum Internet scenario.

**Table:** 

**Algorithm 3** Scalable routing in the quantum Internet	
**Input**: Routing space $${{\mathrm{S}}}_{\mathfrak {R}} \left( N\right) $$ of *N*. **Output**: $${{\mathscr {R}}}_{S} \left( N\right) $$ routing scaling for the quantum network *N*. **Step 1**. Set a critical upper bound $$\partial S^{*} \ge 0$$ on the service rate fluctuation in *N*. Assume that $$\Upsilon \left( U_{swap}\right) $$ is constant for all swapping quantum repeaters (i.e., $$\Upsilon \left( U_{swap}\right) $$ has no impact on routing). **Step 2**. Determine $$R_{j} \left( W_{R_{k} \rightarrow B} \right) $$ and $$R_{i} \left( W_{R_{j} \rightarrow B} \right) $$ in nodes $$R_{j} $$ and $$R_{i} $$ via Algorithm 2. **Step 3**. In $$R_{j} $$, select a neighbour node $$R_{n} $$ that maximizes $$R_{j} \left( W_{R_{k} \rightarrow B} \right) $$ and set it as $$R_{n} =R_{k} $$. In $$R_{i} $$, select a neighbour node $$R_{n} $$ that maximizes $$R_{i} \left( W_{R_{j} \rightarrow B} \right) $$ and set it as $$R_{n} =R_{j} $$. **Step 4**. Update the initial path $${{\mathscr {P}}}^{\left( 0\right) } \left( R_{i} \rightarrow B\right) $$ to path $${{\mathscr {P}}}^{*} \left( R_{i} \rightarrow B\right) $$ with the highest service rate (53) $$W_{R_{i} \rightarrow B} $$ between $$R_{i} $$ and *B*. **Step 5**. Compute $$S_{l=1} \left( R_{i},R_{j} \right) $$ between $$R_{i} $$ and $$R_{j} $$ via (39) as $$S_{l=1} \left( R_{i},R_{j} \right) =S\left( R_{i} \right) +S\left( R_{j} \right) +\xi \left( R_{i},R_{j} \right) $$, where $$S\left( R_{i} \right) $$ and $$S\left( R_{j} \right) $$ are determined via (13), while $$\xi \left( R_{i},R_{j} \right) $$ is as in (40). **Step 6**. If $$\left| \xi \left( R_{i},R_{j} \right) \right| <\partial S^{*} $$, then use $${{\mathscr {R}}}_{d} $$ deterministic routing between $$R_{i} $$ and $$R_{j} $$. **Step 7**. If $$\left| \xi \left( R_{i},R_{j} \right) \right| \ge \partial S^{*} $$, then use $${{\mathscr {R}}}_{a} $$ adaptive routing between $$R_{i} $$ and $$R_{j} $$. **Step 8**. Repeat steps 5–7 for all $$\left\{ R_{i},R_{j} \right\} $$ quantum repeater pairs of path $${{\mathscr {P}}}^{*} \left( R_{i} \rightarrow B\right) $$. **Step 9**. Apply step 4 to find $${{\mathscr {P}}}^{*} \left( R_{S} \rightarrow R_{D} \right) $$ for all $$R_{S} $$ source and $$R_{D} $$ destinations, and repeat step 8 for all $$\left\{ R_{i},R_{j} \right\} $$ quantum repeater pairs of path $${{\mathscr {P}}}^{*} \left( R_{S} \rightarrow R_{D} \right) $$. **Step 10**. Output sets $${{\mathscr {S}}}_{{{\mathscr {R}}}_{d} } ={{\mathscr {S}}}_{{{\mathscr {R}}}_{d,1} } \bigcup \cdots \bigcup {{\mathscr {S}}}_{{{\mathscr {R}}}_{d,{{\mathscr {D}}}} } $$, and $${{\mathscr {S}}}_{{{\mathscr {R}}}_{a} } ={{\mathscr {S}}}_{{{\mathscr {R}}}_{a,1} } \bigcup \cdots \bigcup {{\mathscr {S}}}_{{{\mathscr {R}}}_{a,{{\mathscr {A}}}} } $$, where $${{\mathscr {D}}}$$ and $${{\mathscr {A}}}$$ are the number of subsets of quantum nodes for which $${{\mathscr {R}}}_{d} $$ and $${{\mathscr {R}}}_{a} $$ routing functions can be applied in *N*. Output the $${{\mathscr {R}}}_{S} \left( N\right) $$ scaled routing function for the quantum network *N* as $${{\mathscr {R}}}_{S} \left( N\right) =p_{d} {{\mathscr {R}}}_{d} +p_{a} {{\mathscr {R}}}_{a}$$, where $${{p}_{d}}={\left| {{{\mathscr {S}}}_{{{{\mathscr {R}}}_{d}}}} \right| }/{\left| {{V}_{R}} \right| }$$ and $${{p}_{a}}={\left| {{{\mathscr {S}}}_{{{{\mathscr {R}}}_{a}}}} \right| }/{\left| {{V}_{R}} \right| }$$ are the probabilities of deterministic and adaptive routing in the network, $$p_{a} +p_{d} =1$$, while $$\left| V_{R} \right| =\left| {{\mathscr {S}}}_{{{\mathscr {R}}}_{a} } \right| +\left| {{\mathscr {S}}}_{{{\mathscr {R}}}_{d} } \right| $$ and $$\left| \cdot \right| $$ is the cardinality.	(54)

### Deterministic and adaptive routing

In a $${{\mathscr {R}}}_{d} $$ deterministic routing between $$R_{i} $$ and $$R_{j} $$, the shortest path $${{\mathscr {P}}}^{*} \left( R_{i} \rightarrow R_{j} \right) $$ is fixed such that $$R_{j} $$ is always selected as the neighbouring node of $$R_{i} $$ from the set $${{\mathscr {S}}}_{N} \left( R_{i} \right) $$ of possible neighbours in $$R_{i} $$ (A shortest path is selected with respect to a particular cost function, in our setting the cost function of the path selection is the inverse of $$W_{R_{i} \rightarrow R_{j} } $$). $${{\mathscr {R}}}_{d} $$ deterministic routing is theoretically more compact and faster than adaptive routing $${{\mathscr {R}}}_{d} $$. Practically, this also means that $${{\mathscr {R}}}\left( AB,R_{i} \left( l_{j},l_{k} \right) \right) $$ in (22) is predetermined^114, 115^, regardless of topology and cost function. A straightforward selection for $${{\mathscr {R}}}_{d} $$ is

55 $$\begin{aligned} {{\mathscr {R}}}_{d} \left( R'\right) =\left\{ \begin{array}{ll} f_{N} \left( R'\right) =R_{j},&{}\quad {{\mathrm{if}}} \; R'=R_{i} \\ f_{N} \left( R'\right) =\emptyset ,&{}\quad {{\mathrm{otherwise}}}, \end{array}\right. \end{aligned}$$

where $R'$ is a current node processed by ${{\mathscr {R}}}_{d}$, while function $f_{N} \left( R'\right)$ selects the next neighbour node of $R'$.

In $${{\mathscr {R}}}_{a} $$ adaptive routing between $$R_{i} $$ and $$R_{j} $$, the shortest path $${{\mathscr {P}}}^{*} \left( R_{i} \rightarrow R_{j} \right) $$ is not fixed. The next neighbour $$R_{j} $$ is adaptively selected from set $${{\mathscr {S}}}_{N} \left( R_{i} \right) $$ according to the current network situation. $${{\mathscr {R}}}_{a} $$ adaptive routing requires more resources and computational power than does $${{\mathscr {R}}}_{d} $$. Practically, this also means that $${{\mathscr {R}}}\left( AB,R_{i} \left( l_{j},l_{k} \right) \right) $$ in (22) is not predetermined and depends on the actual topology and cost function of $${{\mathscr {R}}}_{a} $$. Algorithm 2 is a straightforward selection for $${{\mathscr {R}}}_{a} $$ in our setting.

**Figure 3:**
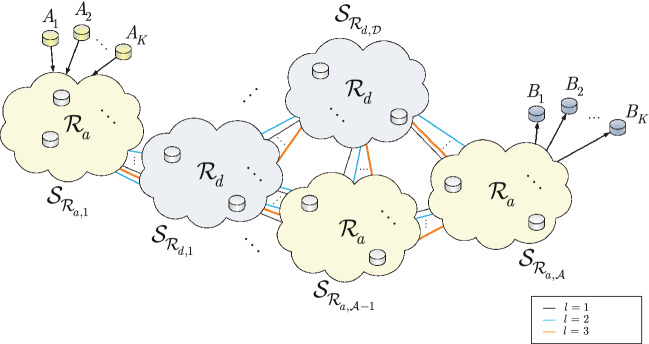
Scaled routing in the quantum Internet. The network is decomposed into subnetworks with ${{\mathscr {R}}}_{a}$ adaptive routing (depicted by yellow clouds) and ${{\mathscr {R}}}_{d}$ deterministic routing (depicted by grey-blue clouds) between the nodes of the subnetwork. Each subset consists of quantum repeaters and entangled connections with heterogeneous entanglement levels.

Figure 3 depicts scaled routing in a quantum Internet setting. The network consists of *K* transmit users, $A_{1},\ldots ,A_{K}$, and *K* receiver users $B_{1},\ldots ,B_{K}$. As the highest service rate path ${{\mathscr {P}}}^{*} \left( A_{i} \rightarrow B_{i} \right)$ is determined between all transmit and receiver users, the quantum repeaters nodes of the quantum network are partitioned into subnetworks ${{\mathscr {S}}}_{{{\mathscr {R}}}_{d} } ={{\mathscr {S}}}_{{{\mathscr {R}}}_{d,1} } \bigcup \cdots \bigcup {{\mathscr {S}}}_{{{\mathscr {R}}}_{d,{{\mathscr {D}}}} }$ with deterministic routing ${{\mathscr {R}}}_{d}$ and ${{\mathscr {S}}}_{{{\mathscr {R}}}_{a} } ={{\mathscr {S}}}_{{{\mathscr {R}}}_{a,1} } \bigcup \cdots \bigcup {{\mathscr {S}}}_{{{\mathscr {R}}}_{a,{{\mathscr {A}}}} }$ with adaptive routing ${{\mathscr {R}}}_{a}$ between the nodes.

### Performance evaluation

#### Service rate

**Figure 4:**
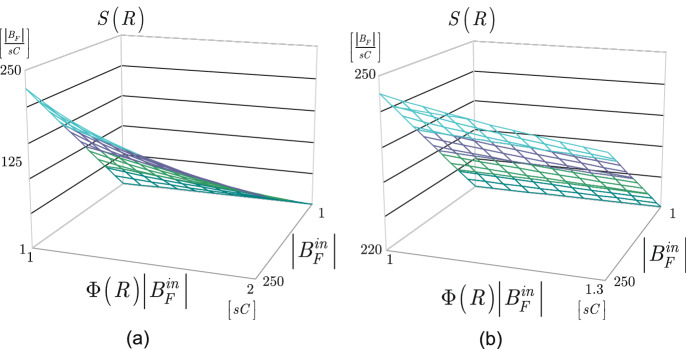
The $S\left( R\right)$ service rate of a quantum repeater *R* with an optical fiber ${{\mathscr {N}}}$ with a standard link loss ${{\mathscr {L}}}\left( {{\mathscr {N}}}\right) \approx 3.3\; {\mathrm{dB}}$. The service rate is depicted in function of the $\left| B^{in}_F\right|$ number of incoming Bell states at a particular fidelity *F* and $\Phi \left( R\right) \left| B^{in}_F\right|$, where $\Phi \left( R\right)$ is evaluated via (25), node efficiency (ratio of outcoming and incoming number of Bell states) ${\eta }_R={\left| B_F\right| }/{\left| B^{in}_F\right| }=0.9$. **a** The values of $\left| B_{F}^{in} \right|$ are set to the range of [0, 250], while $\Phi \left( R\right) \left| B^{in}_F\right|$ is scaled between $\Phi \left( R\right) \left| B^{in}_F\right| \in \left[ 1,2\right]$ *sC* cycles. **b** The values of $\left| B_{F}^{in} \right|$ are set to the range of [0, 250], while $\Phi \left( R\right) \left| B^{in}_F\right|$ is scaled between $\Phi \left( R\right) \left| B^{in}_F\right| \in \left[ 1,1.3\right] \, sC$ cycles.

Assume that *R* is a quantum repeater in *N* with a standard quality optical fiber ${{\mathscr {N}}}$ with a link loss ${{\mathscr {L}}}\left( {{\mathscr {N}}}\right) \approx 3.3\; {\mathrm{dB}}$. In Fig. 4, the $S\left( R\right)$ service rate of quantum repeater *R* is depicted in function of the $\left| B_{F}^{in} \right|$ number of incoming Bell states at a particular fidelity *F* through ${{\mathscr {N}}}$, and in function of $\Phi \left( R\right) \left| B^{in}_F\right|$, where $\Phi \left( R\right)$ is evaluated via (25). The service rate is measured as the $\left| B_{F} \right|$ number of outcoming Bell states per *sC* cycles, where *s* is selected such that $st_{C} =1\,{\hbox {s}}$, while the $\Phi \left( R\right) \left| B^{in}_F\right|$ quantity is measured in *sC* cycles. The values of $\left| B_{F}^{in} \right|$ are set to the range of [0, 250], while $\Phi \left( R\right) \left| B^{in}_F\right|$ is scaled between $\Phi \left( R\right) \left| B^{in}_F\right| \in \left[ 1,2\right]$ *sC* cycles (Fig. 4a) and between $\Phi \left( R\right) \left| B^{in}_F\right| \in \left[ 1,1.3\right]$ *sC* cycles (Fig. 4b), at node efficiency (ratio of outcoming and incoming number of Bell states) ${\eta }_R={\left| B_F\right| }/{\left| B^{in}_F\right| }=0.9$.

#### Computational complexity

Let $\left| V_{R} \right|$ be the total number of quantum repeaters in *N*, with an average number $\left| {{\mathscr {S}}}_{in} \left( R\right) \right|$ of incoming entangled connections per quantum repeater *R*, and with an average number $\left| {{\mathscr {S}}}_{out} \left( R\right) \right|$ of outcoming entangled connections. Then, by utilizing the complexity of a service rate determination^114, 115^, the computational complexity of the routing scaling algorithm is

56 $$\begin{aligned} {{\mathscr {O}}}\left( \left| V_{R} \right| \cdot \left| {{\mathscr {S}}}_{in} \left( R\right) \right| ^{2} \cdot \left| {{\mathscr {S}}}_{out} \left( R\right) \right| \right) . \end{aligned}$$

Assuming that $\left| {{\mathscr {S}}}_{in} \left( R\right) \right| =\left| {{\mathscr {S}}}_{out} \left( R\right) \right|$ in the nodes, the computational complexity is

57 $$\begin{aligned} {{\mathscr {O}}}\left( \left| V_{R} \right| \cdot \left| {{\mathscr {S}}}_{in} \left( R\right) \right| ^{3} \right) . \end{aligned}$$

For a detailed proof, see Section A.1 of the Appendix.

#### Comparison

The computational complexity of the algorithm is compared with the computational complexity of the PG (performance queueing) G/G/1 routing method^115^.

For analytical purposes, we assume a realistic network setting with $\left| {{\mathscr {S}}}_{in} \left( R\right) \right| <\sqrt{\left| V_{R} \right| }$, and for simplicity we set $\left| {{\mathscr {S}}}_{in} \left( R\right) \right| =\left| {{\mathscr {S}}}_{out} \left( R\right) \right|$, with $\left| {{V}_{R}} \right| \in \left[ {{1,10}^{3}} \right]$, and ${{\left| {{{\mathscr {S}}}_{in}}\left( R \right) \right| }^{3}}\in \left[ {{1,10}^{3}} \right]$.

**Figure 5:**
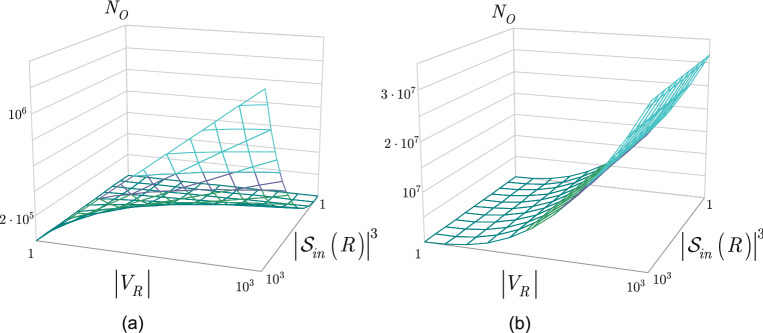
**a** The computational complexity ($$N_{O} $$: number of operations) of the proposed algorithm at a realistic network setting, $$\left| {{\mathscr {S}}}_{in} \left( R\right) \right| <\sqrt{\left| V_{R} \right| }$$, with $$\left| {{\mathscr {S}}}_{in} \left( R\right) \right| =\left| {{\mathscr {S}}}_{out} \left( R\right) \right| $$, in function of $$\left| V_{R} \right| $$ and $$\left| {{\mathscr {S}}}_{in} \left( R\right) \right| ^{3}$$, $$\left| {{V}_{R}} \right| \in \left[ {{1,10}^{3}} \right] $$, $${{\left| {{{\mathscr {S}}}_{in}}\left( R \right) \right| }^{3}}\in \left[ {{1,10}^{3}} \right] $$. **b** The computational complexity of the PG method at a realistic network setting, $$\left| {{\mathscr {S}}}_{in} \left( R\right) \right| <\sqrt{\left| V_{R} \right| }$$, $${\mathscr {O}}( {{\left| V_{R} \right| }^{{5}/{2}\;}})$$ at a particular $$\left| V_{R} \right| $$, $$\left| {{V}_{R}} \right| \in \left[ {{1,10}^{3}} \right] $$, $${{\left| {{{\mathscr {S}}}_{in}}\left( R \right) \right| }^{3}}\in \left[ {{1,10}^{3}} \right] $$.

Figure 5a depicts the resulting complexity of our algorithm, the complexity of the PG routing method is depicted in Fig. 5b.

## Conclusions

Here, we defined a method for routing space evaluation and scalable routing in the quantum Internet. The derived methods utilize the framework of queueing theory along with the characteristics and physical attributes of the quantum Internet. We proved the service rate formulas of quantum repeaters and entangled paths. We defined a method for routing space evaluation to explore the service rates of quantum repeaters and entangled paths of the quantum Internet. Using the results of the routing space exploration, we defined scalable routing for the quantum Internet. The scaled routing function determines the most appropriate routing mechanism for the subnetworks to realize high efficiency and routing in the quantum Internet.

### Ethics statement

This work did not involve any active collection of human data.

## Supplementary information

Supplementary information

## Data Availability

This work does not have any experimental data.
