# Supplementary material for: Routing space exploration for scalable routing in the quantum Internet
Source: Sci Rep. 2020 Jul 17;10:11874. doi: 10.1038/s41598-020-68354-y (PMC7367878; doi:10.1038/s41598-020-68354-y)
Supplement: Supplementary file 1 — Supplementary information [file 41598_2020_68354_MOESM1_ESM.pdf]

Routing Space Exploration for Scalable Routing in the Quantum Internet

Laszlo Gyongyosi[1,2,3,*] **and Sandor Imre**[2]

[1]School of Electronics and Computer Science, University of Southampton, Southampton, SO17 1BJ, UK
[2]Department of Networked Systems and Services, Budapest University of Technology and Economics, Budapest, H-1117 Hungary
[3]MTA-BME Information Systems Research Group, Hungarian Academy of Sciences, Budapest, H-1051 Hungary
[*]gyongyosi@hit.bme.hu

ABSTRACT

The entangled network structure of the quantum Internet formulates a high complexity routing space that is hard to explore. Scalable routing is a routing method that can determine an optimal routing at particular subnetwork conditions in the quantum Internet to perform a high-performance and low-complexity routing in the entangled structure. Here, we define a method for routing space exploration and scalable routing in the quantum Internet. We prove that scalable routing allows a compact and efficient routing in the entangled networks of the quantum Internet.

A Appendix

A.1 Computational Complexity

The proof of the computational complexity is as follows. The computation of the $S(R_i)$ service rate of a particular quantum repeater R_i requires $\mathscr{O}\left(\left|\mathscr{S}_{out}(R)\right|^2\right)$ complexity on average. At $|V_R|$ quantum repeaters, the total computational complexity is

$$\mathscr{O}\left(|V_R|\cdot\left|\mathscr{S}_{out}(R)\right|^2\right). \tag{A.1}$$

Then, the cumulative service rate $S_\Sigma(\mathscr{P}(A\to B))$ for all source A and destination B in the network N is obtainable with a computational complexity

$$\mathscr{O}\left(|V_R|\cdot\left|\mathscr{S}_{in}(R)\right|^2\cdot\left|\mathscr{S}_{out}(R)\right|\right). \tag{A.2}$$

The overall complexity of the algorithm is therefore

$$\mathscr{O}\left(|V_R|\cdot\left|\mathscr{S}_{out}(R)\right|^2\right)+\mathscr{O}\left(|V_R|\cdot\left|\mathscr{S}_{in}(R)\right|^2\cdot\left|\mathscr{S}_{out}(R)\right|\right)=\mathscr{O}\left(|V_R|\cdot\left|\mathscr{S}_{in}(R)\right|^2\cdot\left|\mathscr{S}_{out}(R)\right|\right), \tag{A.3}$$

thus at $\left|\mathscr{S}_{in}(R)\right|=\left|\mathscr{S}_{out}(R)\right|$, the computational complexity is

$$\mathscr{O}\left(|V_R|\cdot\left|\mathscr{S}_{in}(R)\right|^3\right). \tag{A.4}$$

A.2 Notations

The notations of the manuscript are summarized in Table A.1.

Table A.1. Summary of notations.

Notation	*Description*
N	An entangled quantum network, $N=(V,E)$, where V is a set of nodes, E is a set of entangled connections.
A	A source user (quantum node) in the quantum network.

R_i	An i-th quantum repeater, $i = 1, \ldots, q$, where q is the total number of quantum repeaters.
R_h	A previous neighbor of R_i.
R_j	A next neighbor of R_i (towards destination).
l	Level of entanglement.
$\mathrm{L}_l(x,y)$	An l-level entangled connection between quantum nodes x and y.
$d(x,y)_{\mathrm{L}_l}$	Hop-distance at an L_l-level entangled connection between quantum nodes x and y, $d(x,y)_{\mathrm{L}_l} = 2^{l-1}$.
O_C	An oscillator with frequency f_C, $f_C = 1/t_C$, serves as a reference clock.
C	A cycle, with $t_C = 1/f_C$ sec.
sC	Multiple cycles, $st_C = s/f_C$ sec, where s is a nonzero number.
$R_i(l_j, l_k)$	An i-th quantum repeater with incoming entangled connection l_j and outcoming entangled connection l_k.
$\mathscr{S}_{l_{in}}(R_i)$	A set of input entangled connections of quantum repeater R_i, $\mathscr{S}_{l_{in}}(R_i) = (l_{in,1}, \ldots, l_{in,p})$, where p is the total number of input entangled connections.
$\mathscr{S}_{l_{out}}(R_i)$	A set of output entangled connections of quantum repeater R_i, $\mathscr{S}_{l_{out}}(R_i) = (l_{out,1}, \ldots, l_{out,r})$, where r is the total number of output entangled connections.
$S(R_i(l_j, l_k))$	Service rate of R_i at incoming entangled connection l_j and outcoming entangled connection l_k [Bell states per C].
$\Phi(R_i(l_j, l_k))$	Inverse of $S(R_i(l_j, l_k))$ [C cycles].
B	A destination user (quantum node).
$\mathrm{S}_{\mathfrak{R}}$	Routing space of the quantum Internet.
$\mathscr{P}(A_i \to B_i)$	An entangled path between source user A_i and destination user B_i, $i = 1, \ldots, K$, where K is the total number of entangled paths in the quantum network N.
$S_{A_i \to B_i}$	Service rate of entangled path $\mathscr{P}(A_i \to B_i)$.
$S(A_i)$	Service rate of source quantum node A_i.
$S(R_i)$	Service rate of an i-th quantum repeater R_i, $i = 1, \ldots, q$.
$d(x,y)_{\mathrm{L}_l}$	Hop-distance between x and y at an l-level entangled connection $L_l(A,B)$, evaluated as $d(A,B)_{\mathrm{L}_l} = 2^{l-1}$ in the doubling architecture.
q	Total number of quantum repeaters in an entangled path $\mathscr{P}(A_i \to B_i)$, $q = d(A,B)_{\mathrm{L}_l} - 1$.
γ_i	Service rate fluctuation of entangled path $\mathscr{P}(A_i \to B_i)$.
$\gamma(x)$	Service rate fluctuation of a given quantum node x.
Ω_i	Number of available $\mathscr{R}$ routes in the quantum Internet for the entanglement distribution from A_i to B_i.
$\mathscr{S}(\Omega_i)$	Set of Ω_i available routes for a given path $\mathscr{P}(A_i \to B_i)$, $\mathscr{S}(\Omega_i) = \left(\mathscr{R}_1^i, \ldots, \mathscr{R}_{\Omega_i}^i\right)$, where $\mathscr{R}_k^i$ is the k-th available route with $S_{A_i \to B_i}$ and γ_i.
$\mathscr{R}_*^i$	A shortest route for an i-th path $\mathscr{P}(A_i \to B_i)$, $\mathscr{R}_*^i = \max_{\forall k} \mathscr{R}_k^i (S_{A_i \to B_i} + \gamma_i)$.

$\lvert \mathscr{P}(A_i \to B_i) \rvert$	Quantum nodes of an entangled path. In the doubling architecture, $\lvert \mathscr{P}(A_i \to B_i) \rvert = A_i + \sum_{p=1}^{q=d(A,B)_{\mathrm{L}_l}-1} R_p + B_i$, where $d(A,B)_{\mathrm{L}_l} = 2^{l-1}$ is the hop-distance between A and B at an l-level entangled connection $L_l(A,B)$ between A and B.
$S(A(l_k))$	Service rate of source node A with outcoming entangled connection l_k [Bell states per C].
B_F	Entanglement throughput [Bell states per C].
$\lvert B_F^{in}(l_j) \rvert$	Number of incoming entangled states in the input connection l_k of R_i [Number of Bell states].
$\lvert B_F \rvert$	Number of entangled states [Number of Bell states].
$\alpha_k(R_i)$	A ratio for output l_k of R_i.
$\mu(B_F(l_k))$	Average entanglement throughput of output entangled connection l_k of R_i [Bell states per C].
$\mu(B_F(l_j \to l_k))$	Average entanglement throughput of the input entangled connection l_j of R_i [Bell states per C].
$\lvert \mathscr{S}_{l_{in}}(R_i) \rvert$	Cardinality of $\mathscr{S}_{l_{in}}(R_i)$.
$\omega_k(R_i)$	Sum of average entanglement throughput of all incoming entangled connections in R_i [Bell states per C].
$\mu(B_F(A))$	Average output entanglement throughput of source node A [Bell states per C].
$\Pr(\mathscr{P}(A \to B))$	Probability of an entangled path $\mathscr{P}(A \to B)$ between a source A and a target B, $\sum_A \sum_B \Pr(\mathscr{P}(A \to B)) = 1$.
$\mathscr{R}(AB, R_i(l_j, l_k))$	A routing function, $\mathscr{R}(AB, R_i(l_i, l_k)) = \begin{cases} 1, \text{ if } R_i \in \mathscr{P}(A \to B) \\ 0, \text{ otherwise} \end{cases}$. It equals to 1, if quantum repeater $R_i(l_i, l_k)$ is part of the path $\mathscr{P}(A \to B)$, 0 otherwise.
Z	Inverse of $\omega_k(R_i)$ of connection l_j in R_i, $Z = \frac{1}{\omega_k(R_i)}$ [C per Bell states].
$\chi_{in}^2(R_i)$	Coefficient of variation for the Z inverse of incoming entanglement throughput.
$M(R_i(l_k))$	Internal processes of R_i (quantum memory usage, error correction, purification, etc).
$C(M(R_i(l_k)))$	Cycles associated to $M(R_i(l_k))$ [C cycles].
$\chi^2(C(M(R_i(l_k))))$	Coefficient of variation of cycles $C(M(R_i(l_k)))$.
$\varsigma(R_i(l_j, l_k))$	A ratio of incoming and outcoming entanglement throughputs, $\varsigma(R_i(l_j, l_k)) = \frac{\mu(B_F(l_j \to l_k))}{\mu(B_F(l_k))}$.
$\psi_k(R_i)$	Number of residual cycles in R_i [C cycles].
$\theta(R_h(l_k))$	First moment of $C(M(R_h(l_k)))$ in quantum repeater R_h.
$\nu(R_h(l_k))$	Second moment of $C(M(R_h(l_k)))$ in quantum repeater R_h.
$\Pr(R_i(l_j \to l_k))$	Probability that an incoming entangled state from l_j of R_i is distributed through l_k of R_i.
$d(R_i)$	Sum of additional internal and external C cycles related to R_i [C cycles].

$C(\Delta(R_h, R_i))$	External term associated to the $\Delta(R_h, R_i)$ transmission process between nodes R_h and R_i.
$\mathscr{M}$	Quantum memory of quantum repeater R_i.
$\zeta(R_i)$	Cycles of usage of the internal quantum memory $\mathscr{M}$ of R_i [C cycles].
$\left\|\mathscr{M}(R_i(l_j))\right\|$	Number of entangled states received from l_j and stored in the quantum memory $\mathscr{M}$ of R_i.
$\|\mathscr{M}(R_i(l_k))\|$	Number of entangled states readout from the quantum memory $\mathscr{M}$ of R_i and distributed through connection l_k.
$S(\mathscr{P}(A \to B))$	Service rate of an entangled path $\mathscr{P}(A \to B)$ between distant quantum nodes A and B [Bell states per C].
$S_{l=1}(R_S, R_D)$	Service rate between R_S and R_D, where R_S and R_D are quantum repeaters connected by an $l = 1$ level entangled connection $\mathrm{L}_1(x, y)$ [Bell states per C].
$\xi(R_S, R_D)$	Service rate degradation coefficient between source R_S and destination R_D, $\xi(R_S, R_D) \leq 0$.
$W_{A \to B}$	Weighted service rate between A and B.
$\mathscr{P}^*$	An entangled path with the highest weighted service rate $W_{A \to B}$.
∂S^*	An upper bound on the service rate fluctuation, $\partial S^* \geq 0$.
$\Phi_{l=1}(x, y)$	Inverse service rate of between x and y source and target quantum nodes, connected by an $l = 1$ level entangled connection $\mathrm{L}_1(x, y)$ [C per Bell states].
$\Upsilon(U_{swap})$	Service rate decrement in the entanglement distribution caused by the U_{swap} entanglement swapping.
n_{swap}	Number of entanglement swapping operations required for the establishment of an L_l-level entangled connection between distant A and B.
$C\left(R_i^{swap}\right)$	Cycles required by the entanglement swapping an i-th swapping quantum repeater R_i^{swap} [C cycles].
$S_\Sigma(\mathscr{P}(A \to B))$	Cumulative service rate for all source A and destination B in the quantum network N.
$\mathscr{R}_S(N)$	Scaled routing function for the quantum Internet.
$\mathscr{R}_d$	Deterministic routing.
$\mathscr{R}_a$	Adaptive routing.
$\mathscr{S}_{\mathscr{R}_d}$	A set of subnetworks with $\mathscr{R}_d$ deterministic routing, $\mathscr{S}_{\mathscr{R}_d} = \mathscr{S}_{\mathscr{R}_{d,1}} \bigcup \ldots \bigcup \mathscr{S}_{\mathscr{R}_{d,\mathscr{D}}}$, where $\mathscr{D}$ is the number of subsets.
$\mathscr{S}_{\mathscr{R}_a}$	A set of subnetworks with $\mathscr{R}_a$ adaptive routing, $\mathscr{S}_{\mathscr{R}_a} = \mathscr{S}_{\mathscr{R}_{a,1}} \bigcup \ldots \bigcup \mathscr{S}_{\mathscr{R}_{a,\mathscr{A}}}$, where $\mathscr{A}$ is the number of subsets.
p_d	Probability of a $\mathscr{R}_d$ deterministic routing in the quantum Internet, $p_d = \left\|\mathscr{S}_{\mathscr{R}_d}\right\| / \|V_R\|$, where $\left\|\mathscr{S}_{\mathscr{R}_d}\right\|$ is the cardinality of set $\mathscr{S}_{\mathscr{R}_d}$ of subnetworks with $\mathscr{R}_d$ deterministic routing, $\|V_R\| = \|\mathscr{S}_{\mathscr{R}_a}\| + \left\|\mathscr{S}_{\mathscr{R}_d}\right\|$, where $\|\mathscr{S}_{\mathscr{R}_a}\|$ is the cardinality of set $\mathscr{S}_{\mathscr{R}_a}$ of subnetworks with $\mathscr{R}_a$ adaptive routing.
p_a	Probability of a $\mathscr{R}_a$ adaptive routing in the quantum Internet, $p_a = \|\mathscr{S}_{\mathscr{R}_a}\| / \|V_R\|$, where $\|\mathscr{S}_{\mathscr{R}_a}\|$ is the cardinality of set $\mathscr{S}_{\mathscr{R}_a}$ of subnetworks with $\mathscr{R}_a$ adaptive routing, $\|V_R\| = \|\mathscr{S}_{\mathscr{R}_a}\| + \left\|\mathscr{S}_{\mathscr{R}_d}\right\|$, where $\left\|\mathscr{S}_{\mathscr{R}_d}\right\|$ is the cardinality of set $\mathscr{S}_{\mathscr{R}_d}$ of subnetworks with $\mathscr{R}_d$ deterministic routing.

$\mathscr{S}_N(R_j)$	Set of next (toward destination) quantum nodes that share entangled connection with R_j, $R_k \in \mathscr{S}_N(R_j)$.
$R_j\left(W_{R_k \to B}\right)$	Maximal weighted service rate $S_{R_k \to B}$ from the k-th quantum repeater to the destination B evaluated in the j-th node R_j.
$W_{R_n \to B}$	Weighted service rate from R_n to B [Bell states per C].
$V_{R_n \to B}$	Number of quantum repeaters of entangled path $\mathscr{P}(R_n \to B)$ from R_n to B.
$\gamma(R_p)$	Entanglement throughput reduction associated with R_p, and with the cycles $C(\Delta(R_p, R_{p+1}))$ between R_p and R_{p+1}.
$\mathbb{E}\left(R_i\left(W_{R_j \to B}\right)\right)$	Estimation of $R_i\left(W_{R_j \to B}\right)$ in node R_i [Bell states per C].
$\mathscr{P}^{(0)}(R_i \to B)$	An initial path from R_i to B.
$R_i^{(0)}\left(W_{R_j \to B}\right)$	An initial value in R_i for the $W_{R_j \to B}$ weighted service rate from to R_i to B [Bell states per C].
ℓ	Learning rate, $\ell \in [0,1]$.
$W_{R_i \to B}$	Maximized weighted service rate between R_i and B [Bell states per C].
η_R	Node efficiency, $\eta_R = \lvert B_F \rvert \big/ \lvert B_F \rvert$.
$\mathscr{L}(\mathscr{N})$	Link loss [dB].
$\lvert \mathscr{S}_{in}(R) \rvert$	Average number of incoming entangled connections per quantum repeater R.
$\lvert \mathscr{S}_{out}(R) \rvert$	Average number of outcoming entangled connections per quantum repeater R.
